# A method for utilizing automated machine learning for histopathological classification of testis based on Johnsen scores

**DOI:** 10.1038/s41598-021-89369-z

**Published:** 2021-05-10

**Authors:** Yurika Ito, Mami Unagami, Fumito Yamabe, Yozo Mitsui, Koichi Nakajima, Koichi Nagao, Hideyuki Kobayashi

**Affiliations:** Department of Urology, Toho University School of Medicine, 6-11-1, Omori-Nishi, Ota-ku, Tokyo, 143-8541 Japan

**Keywords:** Urology, Testis

## Abstract

We examined whether a tool for determining Johnsen scores automatically using artificial intelligence (AI) could be used in place of traditional Johnsen scoring to support pathologists’ evaluations. Average precision, precision, and recall were assessed by the Google Cloud AutoML Vision platform. We obtained testicular tissues for 275 patients and were able to use haematoxylin and eosin (H&E)-stained glass microscope slides from 264 patients. In addition, we cut out of parts of the histopathology images (5.0 × 5.0 cm) for expansion of Johnsen’s characteristic areas with seminiferous tubules. We defined four labels: Johnsen score 1–3, 4–5, 6–7, and 8–10 to distinguish Johnsen scores in clinical practice. All images were uploaded to the Google Cloud AutoML Vision platform. We obtained a dataset of 7155 images at magnification 400× and a dataset of 9822 expansion images for the 5.0 × 5.0 cm cutouts. For the 400× magnification image dataset, the average precision (positive predictive value) of the algorithm was 82.6%, precision was 80.31%, and recall was 60.96%. For the expansion image dataset (5.0 × 5.0 cm), the average precision was 99.5%, precision was 96.29%, and recall was 96.23%. This is the first report of an AI-based algorithm for predicting Johnsen scores.

## Introduction

The worldwide incidence of infertility problems is about 1 in 7 couples. Over 80% of couples who have regular sexual intercourse and do not use contraception will achieve a pregnancy within 1 year, and approximately 92% can achieve a pregnancy within 2 years^1^. Infertility affects females and males equally. In male infertility, azoospermia is a major problem that prevents a couple from having a child. Azoospermia has two patterns; the first is obstructive azoospermia and the second nonobstructive azoospermia. Obstructive azoospermia implies adequate sperm production but failure in delivery of sperm into the ejaculate because of ductal obstruction. Non-obstructive azoospermia refers to a lack of sperm production^2^.

Types of infertility treatment are intra uterine insemination (IUI), in vitro fertilization (IVF), and intracytoplasmic sperm injection (ICSI). ICSI, which is performed by injecting sperm into an ovum using a thin glass needle, has been found to have a higher rate of fertilization than IVF. The history of ISCI is less than 30 years^3^. Through the use of ICSI, patients with severe oligozoospermia are able to father a baby. However, in the case of patients with azoospermia, it is necessary to obtain testicular sperm from the testis to perform ICSI. After the ICSI technique was performed for the first time, it was reported that ICSI was effective in patients with obstructive and non-obstructive azoospermia^4,5^.

Patients with both types of azoospermia require testicular sperm extraction (TESE) to obtain mature sperms. It comprises conventional TESE (for obstructive type) and microdissection TESE (micro TESE) (for non-obstructive type). Conventional TESE was reportedly performed in 231 patients and micro TESE in 695 patients between April 2014 and March 2015 in Japan^6^.

In addition, we check the condition of testis by collecting a piece of testis tissue in TESE, and the Johnsen score is an effective means of evaluating histological features of the testis^7^. The Johnsen score aims to account for morphological responses to different pathologies affecting testicular cells^8^.

However, histopathological evaluation of the testis is not an easy task and takes much time due to the great complexity of testicular tissue arising from the multiple, highly specialized steps in spermatogenesis, especially in eutherian mammals^9^. In addition, the Johnsen score was first reported 50 years ago and should evolve to suit the modern age.

In recent years, success has been achieved in the application of neural networks and machine learning algorithms in many medical specialties^10–12^. However, particular types of technical skill and mathematical knowledge are required to create deep learning models for use in clinical practice. Such proficiency is still uncommon. Element AI stated that despite the increase in the number of self-reported AI experts worldwide to 36,000, their supply does not meet the demand^13^.

Google (Google Inc., Mountain View, CA) has produced an automated machine learning (AutoML) Vision, which leverages any individual medical images and vast cloud-based processing power^14^. Since there are relatively few physicians with skill in programming, automated deep learning is potentially promising as a platform for expanding the application of deep learning in medical sciences. When applied to classification tasks, machine learning products of this type create a prediction algorithm by automatically matching generic neural network architectures to the imaging dataset provided and fine tuning the network to optimize discriminative performance. Yet, the extent to which health-care professionals without coding experience can achieve the same level of performance as expert deep learning engineers with the support of automated deep learning remains unclear^15^. However, other research groups have already reported on the utility of an automated deep learning approach in distinguishing medical images^16,17^.

In this study, we created a computer vision algorithm for classifying Johnsen scores using Google cloud AutoML Vision.

## Results

**Figure 1:**
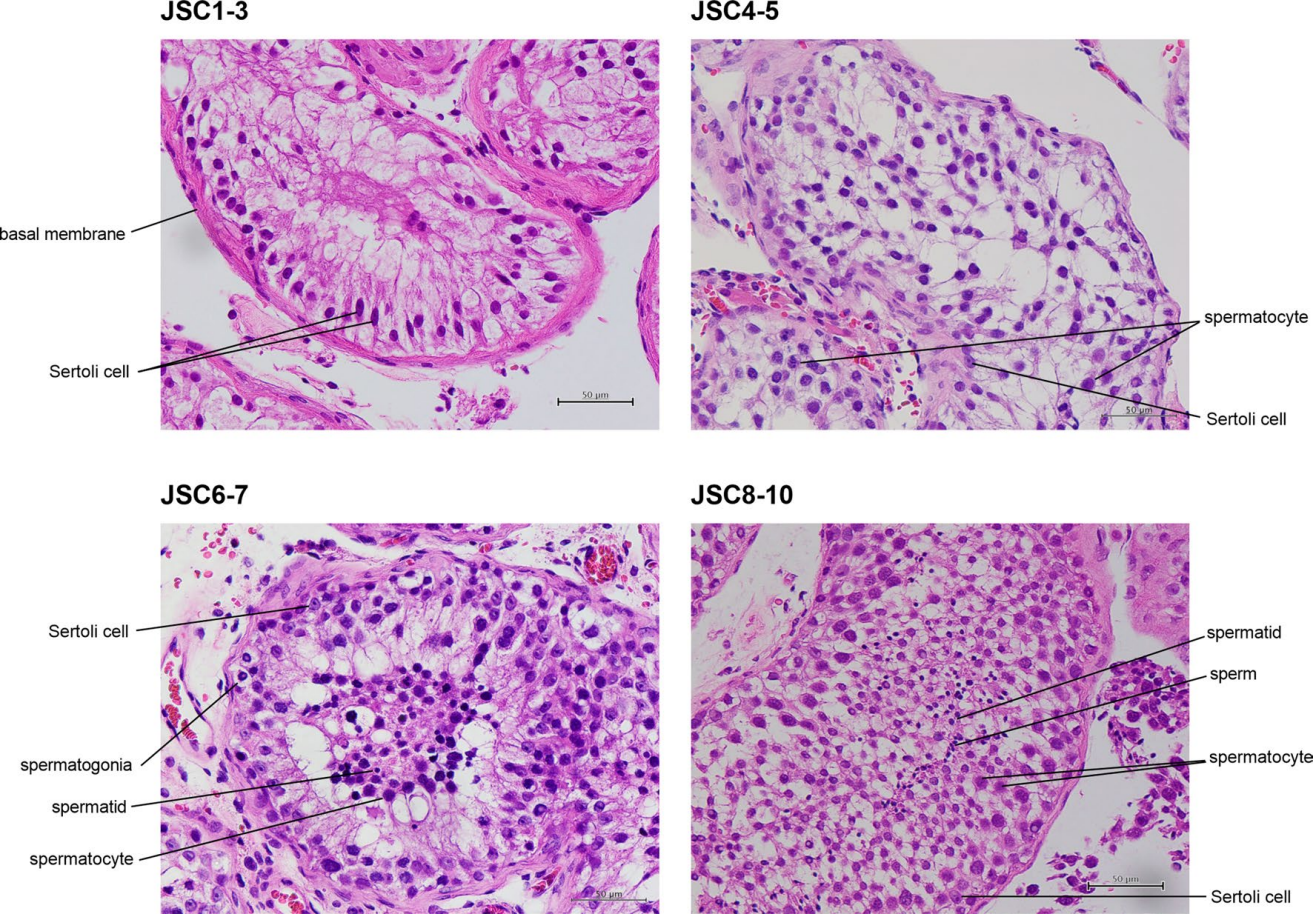
Pathological images for JSC 1–3, 4–5, 6–7, and 8–10. All images are stained with haematoxylin eosin and ×400 magnification. JSC 1–2 do not contain any germ cells and JSC 3 includes spermatogonia, with no spermatocytes. JSC 4–5 include few or many spermatocytes, with no spermatids. JSC 6–7 include few or many spermatids, with no sperms. JSC 8–10 include few or many sperms in a seminiferous tubule.

**Figure 2:**
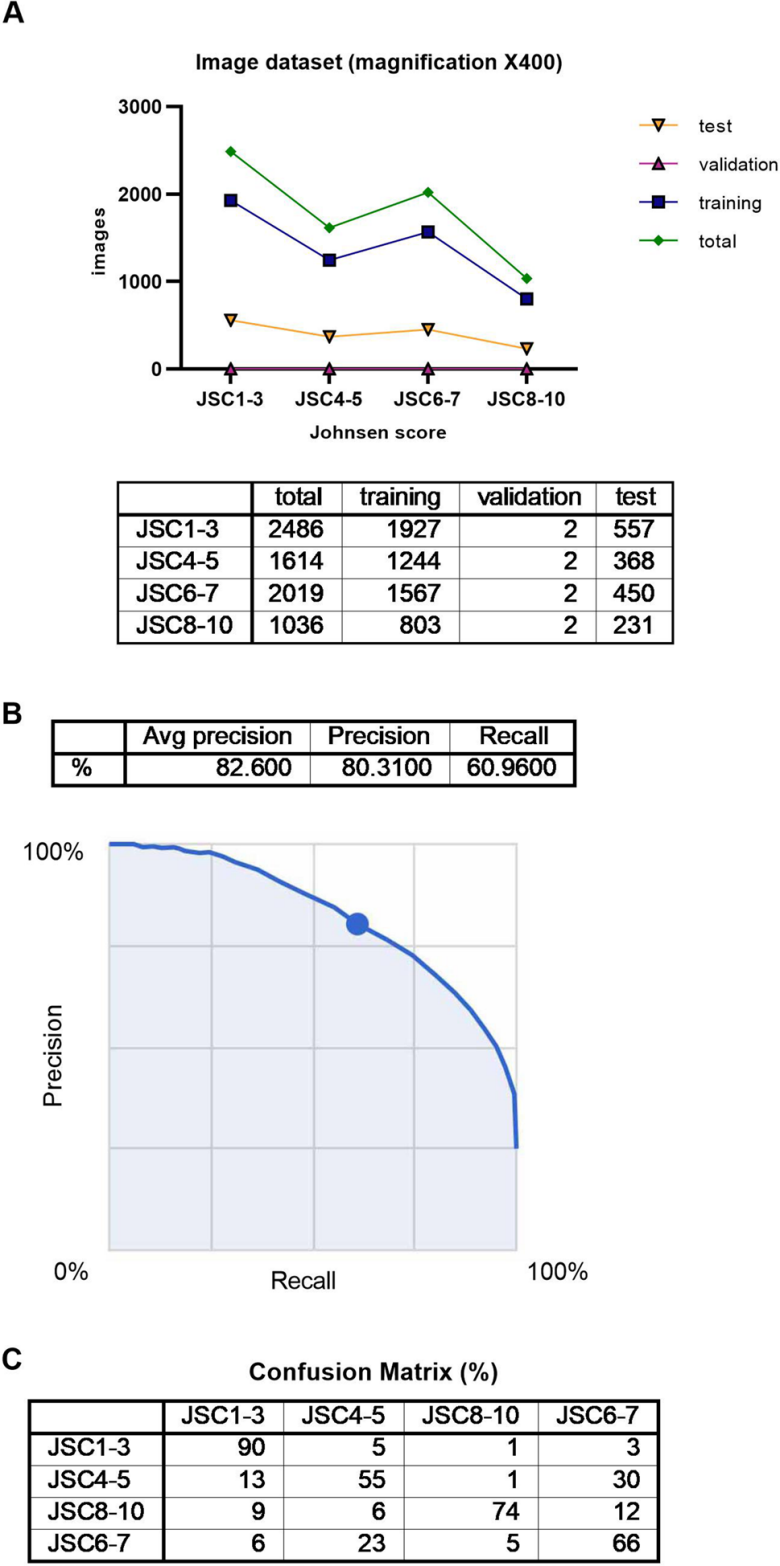
(**A**) Summary of image dataset (magnification ×400). (**B**) Algorithm performance using Google Cloud AutoML Vision, Average precision recall curve for image dataset, magnification ×400. (**C**) Confusion matrix for image dataset, magnification ×400.

An image dataset of 7155 magnification images was generated (400×). A selection of these images for each label can be seen in Fig. 1. The label for a Johnsen score of 1–3 included 2486 images. The training set images, validation set images, and test set images included 1927, 2 and 557 images, respectively. The label for a Johnsen score of 4–5 included 1614 images. The training set images, the validation set images, and the test set images accounted for 1244, 2, and 368 images, respectively. The label for a Johnsen score of 6–7 included 2019 images. The training set images, the validation set images, and the test set images accounted for 1567, 2, and 450 images, respectively. The label for a Johnsen score of 8–10 included 1036 images. The training set images, the validation set images, and the test set images accounted for 803, 2, and 231 images, respectively (Fig. 2A).

The average precision (positive predictive value) of the algorithm was 82.6%, precision was 80.31%, and recall was 60.96% based on automated training and testing by the Google Cloud AutoML Vision in Fig. 2B. The precision recall curves were generated for each individual label as well as for the algorithm overall. We adopted a threshold value of 0.5 to yield balanced precision and recall.

A confusion matrix is shown in Fig. 2C. We found that the label for a Johnsen score of 4–5 was most confused as a Johnsen score of 6–7, at 30%. On the other hand, the frequency of the label for a Johnsen score of 6–7 being confused as a Johnsen score of 4–5 was 23%.

**Figure 3:**
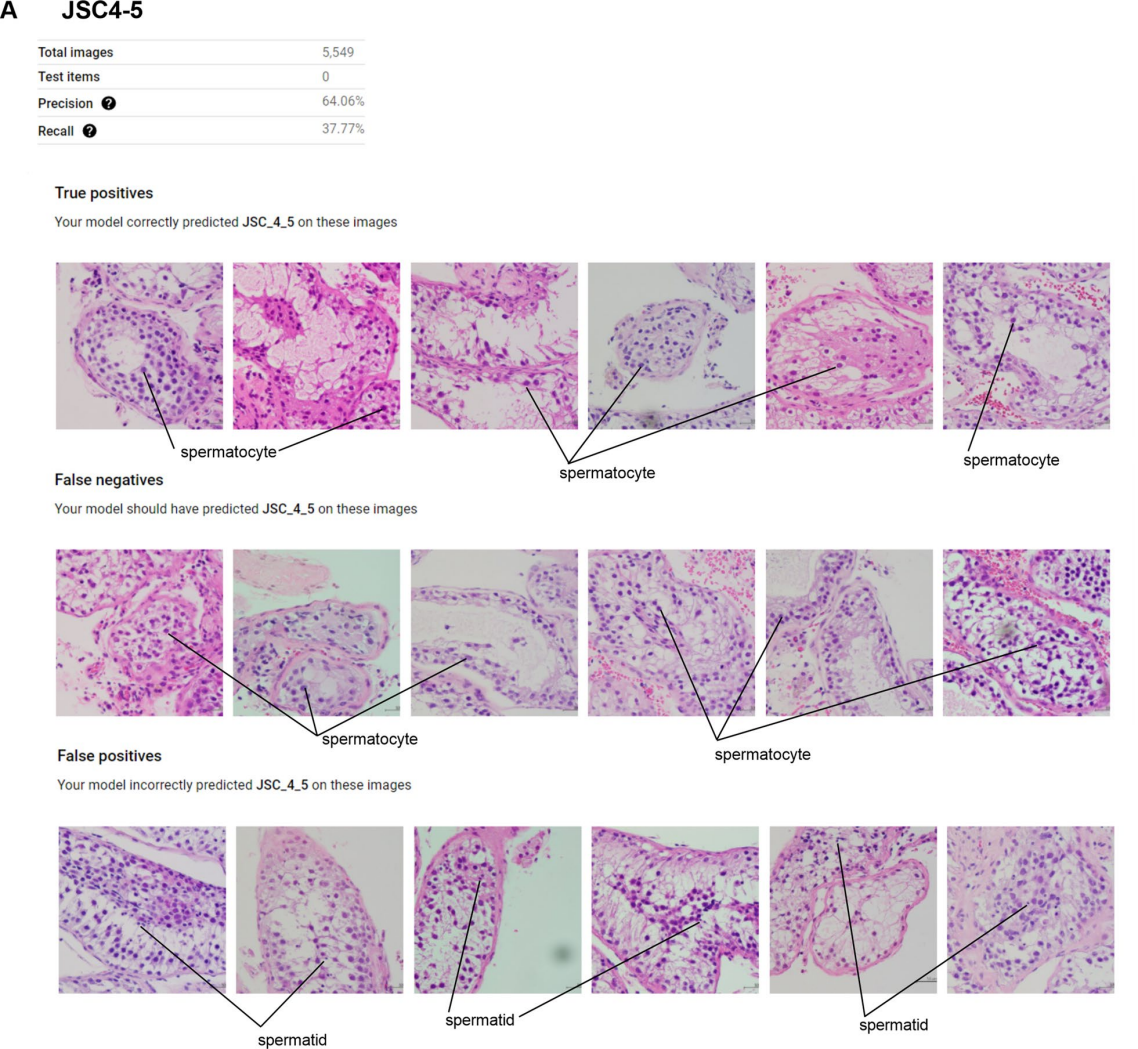
Features of true positives, false negatives, and false positives for JSC4-5.

The precision and recall for a Johnsen score of 4–5 were 64.06% and 37.77%, respectively. True positives are defined as images for which our model correctly predicted a Johnsen score of 4–5. False negatives are defined as images for which our model should have predicted a Johnsen score of 4–5 and false positives are defined as images for which our model incorrectly predicted a Johnsen score of 4–5. In the case of false positives, our model recognized images with a Johnsen score of 4–5 as those with a Johnsen score of 6–7 (Fig. 3).

**Figure 4:**
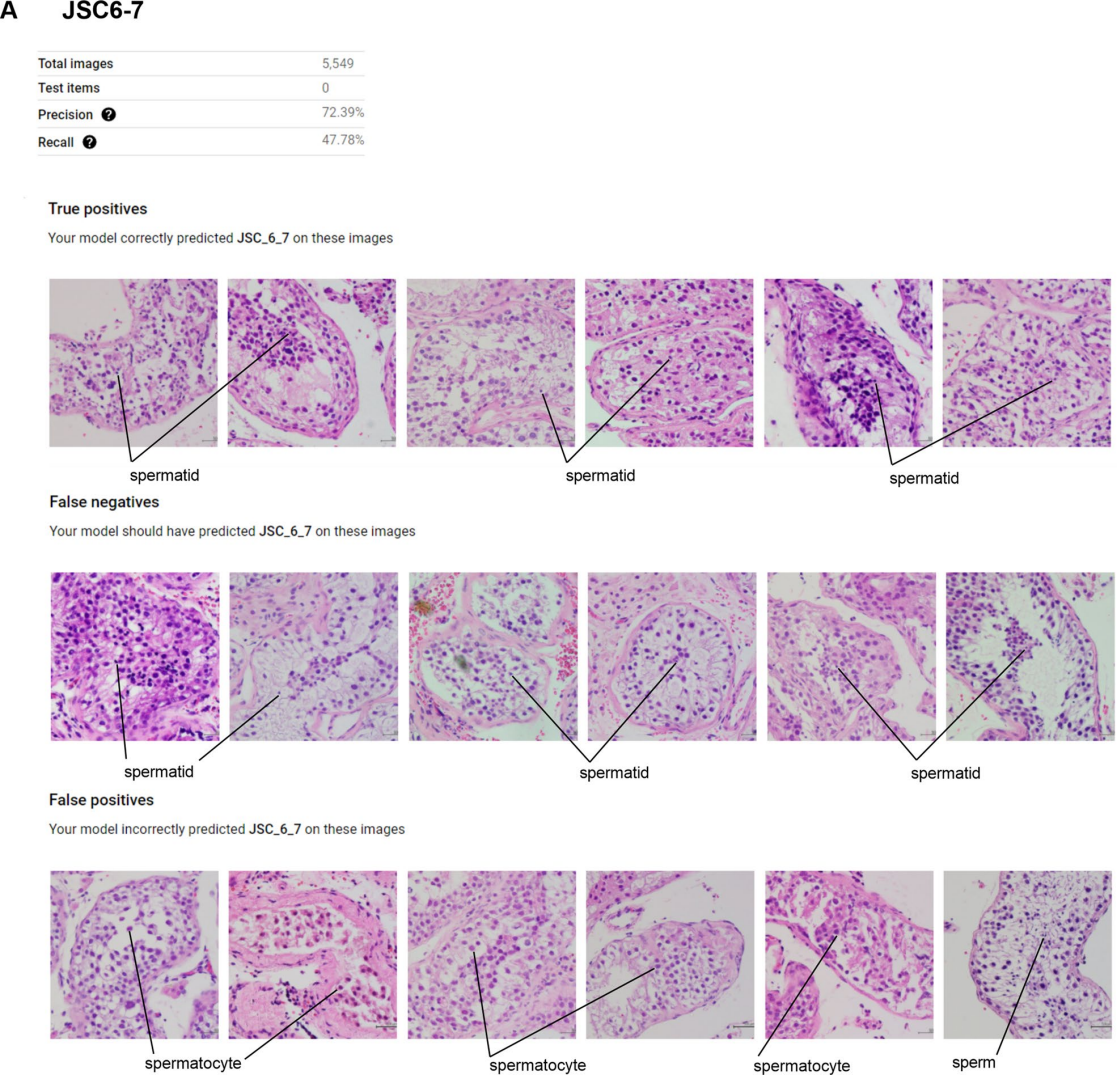
Features of true positives, false negatives, and false positives for JSC6-7.

The precision and recall for a Johnsen score of 6–7 were 72.39% and 47.78%, respectively. Regarding false positives, our model recognized images with a Johnsen score of 6–7 as a Johnsen score of 4–5 or 8–10 (Fig. 4).

**Figure 5:**
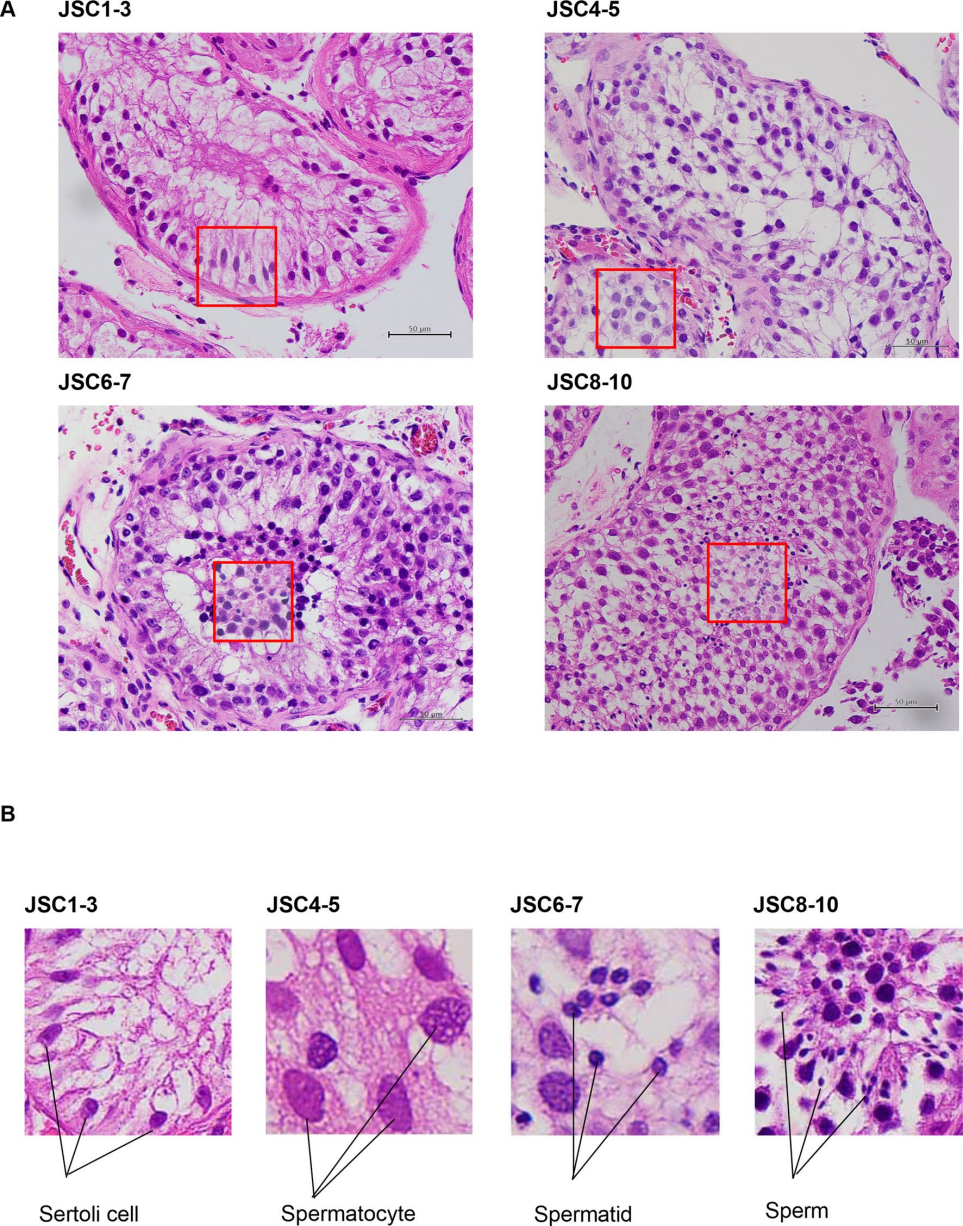
Expanded pathological images for JSC1-3, 4–5, 6–7, and 8–10. Red squares are cut out from pathological image (×400) to determine Johnsen score. (**B**) Characteristics of JSC 1–3, 4–5, 6–7, and 8–10.

**Figure 6:**
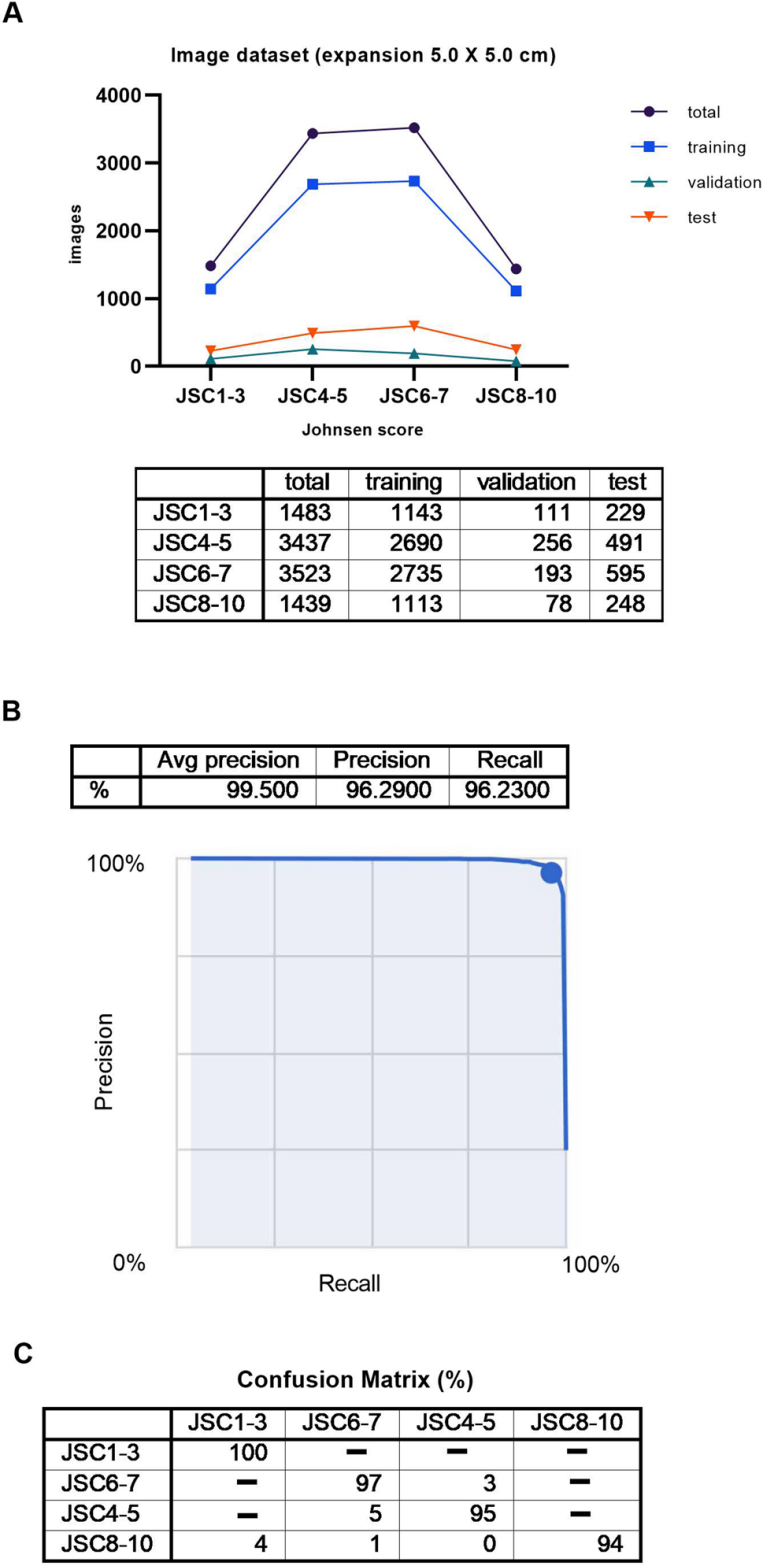
(**A**) Summary of image dataset (expansion, 5.0 × 5.0 cm) (**B**) Algorithm performance using Google Cloud AutoML Vision, Average precision recall curve for image dataset, expansion, 5.0 × 5.0 cm (**C**) Confusion matrix for image dataset, expansion, 5.0 × 5.0 cm.

An image dataset of 9822 expansion images was generated (5.0 × 5.0 cm). A selection of the cut out images (red squares) for each label can be seen in Fig. 5A,B. The label for a Johnsen score of 1–3 included 1483 images. The training set images, the validation set images, and the test set images accounted for 1143, 111, and 229 images, respectively. The label for a Johnsen score of 4–5 included 3437 images. The training set images, the validation set images, and the test set images accounted for 2690, 256, and 491 images, respectively. The label for a Johnsen score of 6–7 included 3523 images. The training set images, the validation set images, and the test set images accounted for 2735, 193, and 595 images, respectively. The label for a Johnsen score of 8–10 included 1439 images. The training set images, the validation set images, and the test set images accounted for 1113, 78, and 248 images, respectively, as shown in Fig. 6A.

The average precision (positive predictive value) of the algorithm was 99.5%, precision was 96.29%, and recall was 96.23% based on automated training and testing by the Google Cloud AutoML Vision as shown in Fig. 6B. The precision recall curves were generated for each individual label as well as for the algorithm overall. We adopted a threshold value of 0.5 to yield balanced precision and recall.

A confusion matrix is shown in Fig. 6C. We found that the label for a Johnsen score of 4–5 was most confused as a Johnsen score of 6–7, at 5%. On the other hand, the frequency of confusing a label for a Johnsen score of 6–7 as a Johnsen score of 4–5 was 3%. In addition, the label for a Johnsen score of 8–10 was confused as a Johnsen score of 1–3, at 4%.

We have given the results for all statistical analyses for the image dataset of 7155 magnification images (400×) and image dataset of 9822 expansion images (5.0 × 5.0 cm) in the supplement (see supplementary data online).

## Discussion

The biggest advantage of Google Cloud AutoML Vision is ease of creating an AI model using a data set with no need for coding. In order to manually create an AI model with an algorithm developed specifically for each study, engineers expert in deep learning would be required. The code-free deep learning approach that we adopted has the potential to improve access to deep learning for clinicians^18^. From now on, we hope that many clinicians in numerous departments will establish AI models by this approach and use them in clinical practice.

We have described the development of an AI-based algorithm for evaluating Johnsen scores combining original images (400×) and expansion images (5.0 cm × 5.0 cm), and it achieved high accuracy. This is the first report of an algorithm that can be used for predicting Johnsen scores without having to rely on pathologists. In addition, we were able to create it through an automated machine learning approach requiring no coding experience.

Other research groups have already reported on medical image classification or otoscopic diagnosis performed using an automated deep learning approach with no coding^15,19^. We consider that an AI-based algorithm would also be suitable for quantification such as that in Gleason grading for prostate cancer. Gleason grading is an established scoring system for prostate cancer that is widely used by pathologists worldwide^20^. In fact, the development of a medical grade AI-based algorithm for evaluating prostate core needle biopsies using Gleason scores has already been reported^21^.

In cases of severe male infertility, which have been categorized as non-obstructive azoospermia or obstructive azoospermia, TESE is needed for obtaining testicular sperm. Non-obstructive azoospermia refers to a lack of sperm production, whereas obstructive azoospermia implies adequate sperm production but obstruction of the ductal system.

Non-obstructive azoospermia can be the consequence of a number of genetic or environmental conditions including Klinefelter syndrome, Y chromosome micro deletions, cryptorchidism, hypogonadism, varicoceles, mumps orchitis, chemotherapy or radiation. Additionally, testicular sperm may provide superior outcomes over epididymal or ejaculated sperm in properly selected cases^22^.

In TESE, seminiferous tubules are easily extruded using light manual pressure. A piece of tissue is sent to the pathologist to determine the Johnsen score, another piece is teased on a sterile slide with a drop of human tubal fluid or sperm wash media and examined under light microscopy. If sperm are seen, more tissue is taken from this area and given to the embryologist for use in ICSI. In micro TESE, the surgeon’s non-dominant hand holds the split testicle while the dominant hand carefully and systematically dissects the seminiferous tubules under high-power magnification^23^. However, a lower percentage of sperm is retrieved with micro TESE than conventional TESE so it is important to examine testis condition using a small piece of testis tissue.

A standardized, reproducible, and objective grading system depends on a number of features. Commonly, an assessment of morphometric features (tubular diameter and basement membrane thickness) and/or cellular features (number of cells and types of cells seen in each field) is used to create a quantitative description of testis biopsy. The score in the Johnsen scoring system, the most commonly used method for scoring testicular biopsy, is predicted on the premise that increasing degrees of testicular damage result in successive depletion of cell types from most to least mature. Tubules are thereby scored on a scale from 1 to 10, where 10 represents intact spermatogenesis, while a score of 1 indicates the absence of all germ cells^7^. The ultimate importance of a scoring system depends on its ability to differentiate clinically meaningful conditions within the testis, such as maturation arrest, from hypo spermatogenesis with contributing partial epididymal obstruction. However, pathologists need much experience to make histopathological evaluations of the testis and there is a growing shortage of pathologists worldwide^24^. Therefore, we considered that an AI-based tool could provide major support to pathologists in their evaluation of Johnsen scores, in place of the traditional Johnsen scoring system.

In this study, we devised a means of obtaining images of testis tissues for use with an AI algorithm. We found that 400× magnification of a seminiferous tubule in an image would be adequate. We also found that it is difficult to distinguish between Johnsen scores of 4–5 and 6–7 for typical images obtained.

It has been reported that allocation of images is random in Google Cloud AutoML Vision. When we uploaded nine images in a single upload, training and test set images were placed without validation set images. We did not know the reason for validation set images not being placed. Later, we found that more than ten images per upload were needed to deploy the training, validation, and test set images randomly.

For the 400× magnification images dataset, we found that the average precision of the algorithm was 82.6%. However, we found that accuracy was lower for Johnsen scores of 4–5 and 6–7—55% and 66%, respectively, and considered that it is difficult to distinguish between spermatocytes and spermatids when magnification of the field is 400×. Therefore, we thought of a way of improving accuracy for Johnsen scores of 4–5 and 6–7.

We cut out 5.0 cm square pieces from the 400× magnification images and found that Johnsen scores could be determined for such a small area. This use of the algorithm with this expansion image dataset resulted in improving average precision to 99.5%. The precision for Johnsen scores of 4–5 and 6–7 improved to 95% and 97%, respectively. However, we realized that cut outs had to be made in a special way to determine the Johnsen score. They had to be designed to contain the parts of images that a human would consider to be the most clinically relevant in making a classification, to avoid reducing the validity of AI-tool model. Therefore, we were careful to ensure that cells that were morphologically the same were included in each 5.0 cm square cut out from 400× magnified images of seminiferous tubules.

Regarding observer bias, humans tend to see what they want to see or expect to see when observing objects. The subjectivity of the annotator who cuts out the squares will inevitably be reflected in the areas cut out for expansion. As a measure against such bias, we made definite guidelines for making decisions on areas to be cut out for individual Johnsen score ranges. For Johnsen scores of 1–2, the basal membrane of seminiferous tubules and/or Sertoli cells had to be included in the 5.0 × 5.0 cm square. For a Johnsen score of 3, Sertoli cells and/or spermatogonia had to be included. For Johnsen scores of 4–5, spermatocytes had to be included. For Johnsen scores of 6–7, spermatids had to be included and for Johnsen scores of 8–10, sperms had to be included in the 5.0 × 5.0 cm square.

The guidelines also stated that two persons (I·Y and U·M) had to cut out the 5.0 × 5.0 cm squares from the histopathology images to magnify characteristic areas for determining Johnsen scores. In addition, a third person (K·H) had to check all the images and upload them to Google Cloud AutoML Vision. However, even with the above guidelines for determining cut-out areas checking images and uploading them, we cannot exclude observer bias completely.

Furthermore, the accuracy of the AI-based algorithm for evaluating Johnsen scores would change, depending on the quality of tissue, differences in tissue fixation methods, differences in microscopes, differences in digital cameras, differences in how individual pathologists carry out scoring, and differences in training sets. Differences in testis tissue fixation methods in particular would have the potential to produce differences in the average precision of an AI-based algorithm for evaluating Johnsen scores. Therefore, when other research groups establish their own AI-based algorithms for evaluating Johnsen scores in the future, the algorithm we have created in this study could be a benchmark for comparing models. We are sharing the dataset of all images used in this research as well as the details of the process for using Google Cloud AutoML Vision. Hereafter, we will refine our AI-based algorithm for evaluating Johnsen scores based on our results and through its use in clinical practice.

In conclusion, we created an AI-tool model for classification according to Johnsen scores with no coding experience. Through the use of this model, classification using Johnsen scores should become more widespread in clinical practice.

## Conclusion

We describe the development of an AI-based algorithm for evaluating Johnsen scores combining original images and expansion images. We found that the algorithm achieved relatively high accuracy for the initial dataset. Although the expansion dataset showed very high accuracy, it has the limitation of potential observer bias. This is the first report of an AI-based algorithm for predicting Johnsen scores that does not have to rely on pathologists. We were able to create it using an automated deep learning approach requiring no coding experience. An accurate and convenient AI-based algorithm for determining Johnsen scores will be an important tool for pathologists and urologists treating male infertility worldwide. So far, Johnsen scores have been determined by pathologists but with our AI-based algorithm, they can be automatically determined without pathologists being present. It also allows us to obtain results more quickly than before.

AI has the potential for bringing about major changes in the field of reproductive medicine in the near future. We believe that this article shows novel scientific and clinical features and will promote the use of AI systems in reproductive medicine.

In this study, we created a computer vision algorithm for classifying Johnsen scores using Google cloud AutoML Vision. This algorithm has the potential for use by pathologists or urologists treating male infertility and could be beneficial in remote areas and developing countries in evaluating Johnsen scores for timely referral.

## Methods

### Study population

From January 2010 to December 2019, 275 patients with obstructive or non-obstructive azoospermia who underwent testicular sperm extraction (TESE) were included in this study. In detail, 248 patients underwent micro TESE and 25 patients underwent conventional TESE. Testis biopsy was performed in 2 patients. The study protocol was approved by the Ethics Committee of Toho University Omori Medical Center (approval No. M20103). All methods were performed in accordance with the relevant guidelines and regulations as well as with the Declaration of Helsinki. We could not obtain individual consent from all patients. The presented study design was accepted by the ethics committee on the condition that a document that declares an opt-out policy by which any potential patients and/or their relatives could refuse to be included in this study was uploaded to the website of the Toho University Omori Medical Center.

### Histological analyses

Small pieces of testicular tissue were obtained from the lateral testis for histopathological examination to determine its structural condition. Testis tissue was fixed in formalin solution for at least 30 min, submerged in formaldehyde and embedded in paraffin. A microtome was used to obtain 4 µm thick slices, which were mounted on appropriate glass microscope slides for analysis. Hematoxylin–eosin staining was used to facilitate adequate visualization of the spermatogenic cells. On microscopic evaluation, a sample was considered satisfactory if at least 10–30 seminiferous tubules were visible for cell counting. We used glass microscope slides from 264 of the 275 patients for this study. Those from 11 patients were excluded because a Johnsen score could not be determined. We used 238, 24, and 2 glass microscope slides from micro TESE, conventional TESE, and testis biopsy, respectively.

To obtain the Johnsen score, slides were examined under an optical microscope (magnification, 100× and 400×). A score was assigned for each tubule counted. The number of tubules with a given score was multiplied by the score. The result was summed across different scores and then divided by the number of evaluated tubules, giving the final Johnsen score. The final score was decided by at least two pathologists in Omori Hospital, School of Medicine, Toho University.

### Johnsen score

For the evaluation of the testis, we used the criteria formulated by Johnsen^7^. Johnsen scores use a ten-point scoring system for quantifying spermatogenesis according to the profile of the cells encountered along the seminiferous tubules. A Johnsen score of 10 indicates maximum spermatogenesis activity, whereas a score of 1 indicates complete absence of germ cells.

We defined four specific labels for classifying clinical cases based on Johnsen scores from 1 to 10. The four labels were for Johnsen scores of 1–3, 4–5, 6–7, and 8–10. Johnsen scores 1 and 2 do not contain any germ cells and Johnsen score 3 contains only spermatogonia as germ cells, a Johnsen score of 4–5 includes spermatocytes, a Johnsen score of 6–7 includes spermatids and a Johnsen score of 8–10 includes mature sperms. In our original categories, Johnsen scores of 1–3, 4–5, 6–7, and 8–10 accounted for 117, 44, 36, and 67 cases, respectively, total 264 cases.

### Histopathology images (data source)

Regarding the numbers of pathological glass slides per a patient for the 264 patients, 214 patients had one slide, 15 patients had 2 slides, 30 patients had 3 slides, 1 patient had 4 slides, 2 patients had 6 slides, 1 patient had 9 slides, and 1 patient had 12 slides. The average number of slides per patient was 1.4.

We obtained histopathology images for the testis using a BX43 microscope (magnification, 400×) (Olympus, Japan) and a digital camera DP27 (Olympus, Japan). Images were 1224 × 960 or 2448 × 1920 pixels in size and saved as jpg files. We obtained 21.2 histopathology images per case from Johnsen scores of 1–3, 39.8 histopathology images per case from Johnsen scores of 4–5 and 6–7, and 12.0 histopathology images per case from Johnsen scores of 8–10, referring to the raw Johnsen scores determined by pathologists. We counted 10–50 seminiferous tubules on one pathological glass slide to obtain histopathology images.

In addition, we took cut outs of the histopathology images (5.0 × 5.0 cm) to magnify characteristic areas for determining Johnsen scores within seminiferous tubules using Adobe Photoshop Elements 2020 (Adobe Inc., USA).

Two persons (IY and UM) performed all processes for taking histopathology images of the testis using a microscope based on pathologist reports and cutting out histopathology images using Adobe Photoshop Elements.

After these two persons took the histopathology images of the testis, KH checked all the images and uploaded them to Google Cloud AutoML Vision. Since the processes of taking images and uploading them are described in detail, the results we obtained are reproducible.

We share the raw uncompressed image files at: jsc_classifier: an image dataset of 7155 magnification images (400×) https://console.cloud.google.com/storage/browser/jsc_classifier/jsc_classifier_part2: an image dataset of 9822 expansion images (5.0 × 5.0 cm) https://console.cloud.google.com/storage/browser/jsc_classifier_part2.

### Annotation and algorithm generation

We used Google Cloud AutoML Vision in Google Cloud Platform (GCP) (Google Inc.) for this research. Images were uploaded to the Google Cloud AutoML Vision platform as jpg images, not CSV files. Uploaded images were saved to Google Cloud Storage in GCP. We did not use any programming to upload images to the Google Cloud AutoML Vision platform. We defined four labels. Label 1 included Johnsen scores from 1 to 3 and label 2 included Johnsen scores of 4 and 5. Label 3 included Johnsen scores of 6 and 7 and label 4 included Johnsen scores from 8 to 10. All images were divided according to the four labels. A single label classifier architecture was utilized. All of this process was performed by one physician (KH).

We have explained how to use Google Cloud AutoML Vision (see supplementary information: Methods online).

### Artificial neural network (ANN) programming and training

Using the Google Cloud AutoML Vision platform, the training set images, the validation set images, and the test set images were randomly selected from the dataset automatically. The training set images, the validation set images and the test set images were independent of each other. We had to have all of the image types (training set images, validation set images and the test set images) to perform the learning process for the algorithm. The number of images selected for the test set was proportional to the number in the training set. The algorithm was naïve to the images in the training set. Eight nodes (1 h) were utilized to train the algorithm on the AutoML Vision cloud-based graphical processing units. A single label image classification architecture was utilized.

### Statistical analysis

The code for Google Cloud AutoML Vision platform has not been made publicly available by Google, the company responsible for its development.

AutoML Vision provided metrics that are commonly used by the AI community. These are precision (positive predictive values) and recall (sensitivity) for the stated threshold and area under the curve (AUC). We have also provided confusion matrices for each model, which cross-reference the true labels against those predicted by the deep learning model^15^.

Using extracted binary diagnostic accuracy data, we constructed contingency tables showing calculated specificity at the threshold of 0.5. The contingency tables showed true-positive, false-positive, true-negative, and false-negative results.

### Data sharing

KH has ownership for the image data used with Google Cloud AutoML Vision and it can be utilized in other research by only paying the download cost to Google. The data collected during this study is patient data obtained with the Ethical Committee’s approval and cannot be shared.

## Supplementary Information

Supplementary Information 1.

Supplementary Information 2.
