# Supplementary material for: A method for utilizing automated machine learning for histopathological classification of testis based on Johnsen scores
Source: Sci Rep. 2021 May 10;11:9962. doi: 10.1038/s41598-021-89369-z (PMC8107178; doi:10.1038/s41598-021-89369-z)
Supplement: Supplementary file 1 — Supplementary Information 1. [file 41598_2021_89369_MOESM1_ESM.pdf]

A method for utilizing automated machine learning for histopathlogical classification of testis based on Johnsen scores

Yurika Ito[1], Mami Unagami[1], Fumito Yamabe[1], Yozo Mitsui[1], Koichi Nakajima[1], Koichi Nagao[1], Hideyuki Kobayashi[1,*]

[1] Department of Urology, Toho University School of Medicine, Tokyo, Japan

[*]To whom correspondence should be addressed: Department of Urology, Toho University School of Medicine, Tokyo, Japan, 6-11-1, Omori-Nishi, Ota-ku, Tokyo 143-8541, Japan

TEL +81-3-3762-4151 FAX +81-3-3768-8817

E-mail: hideyukk@med.toho-u.ac.jp

Supplementary data

All statistical analyses for JSC_classifier: an image dataset of 7155 magnification images (X400) and JSC_classifier_Part2: an image dataset of 9822 expansion images (5.0 X 5.0 cm)

JSC_classifier: an image dataset of 7155 magnification images (X400)

The average precision (positive predictive value) of the algorithm was 82.6%, precision was 80.31%, and recall was 60.96% for all labels.

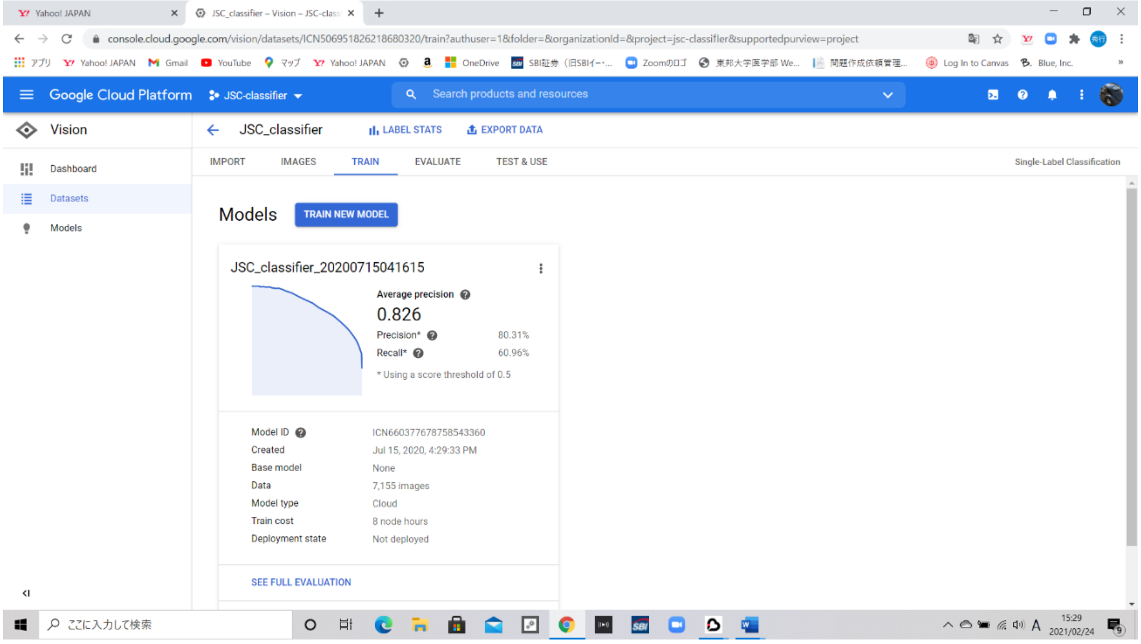

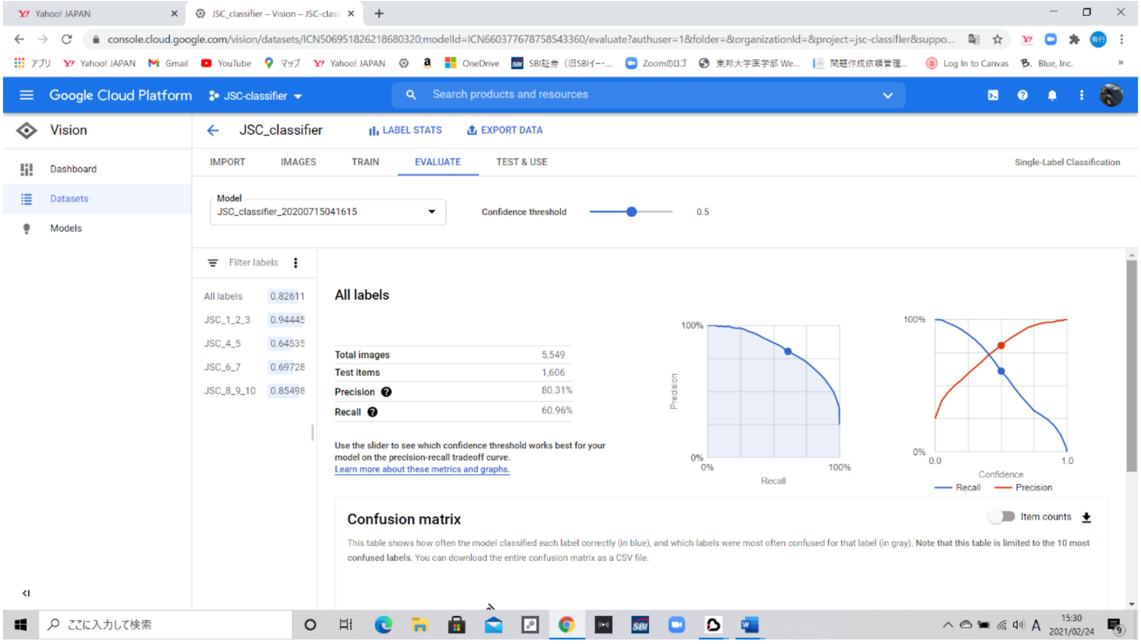

Confusion matrix

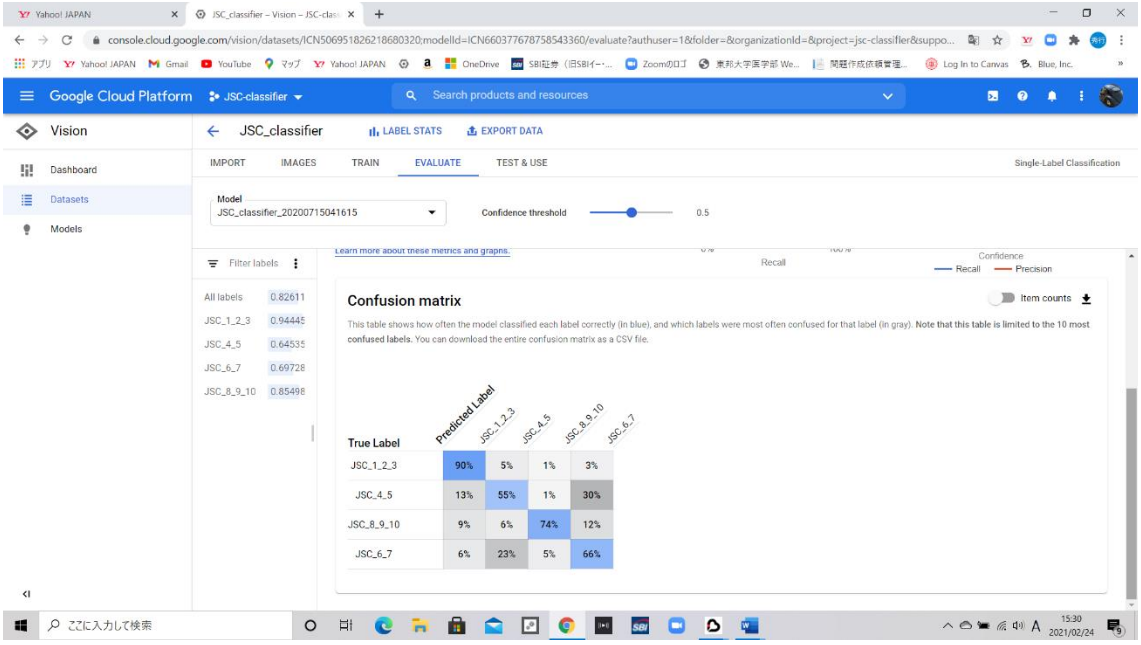

The precision was 88.74% and recall was 84.92% for a Johnsen score of 1-3. True positive, false negative, and false positive images are indicated.

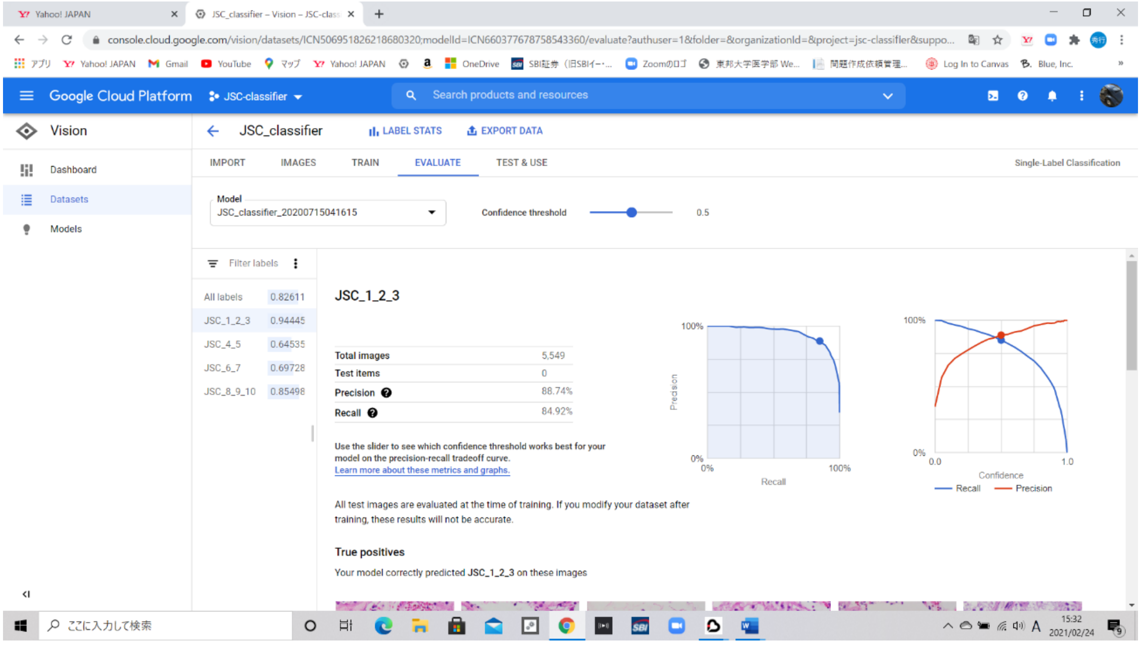

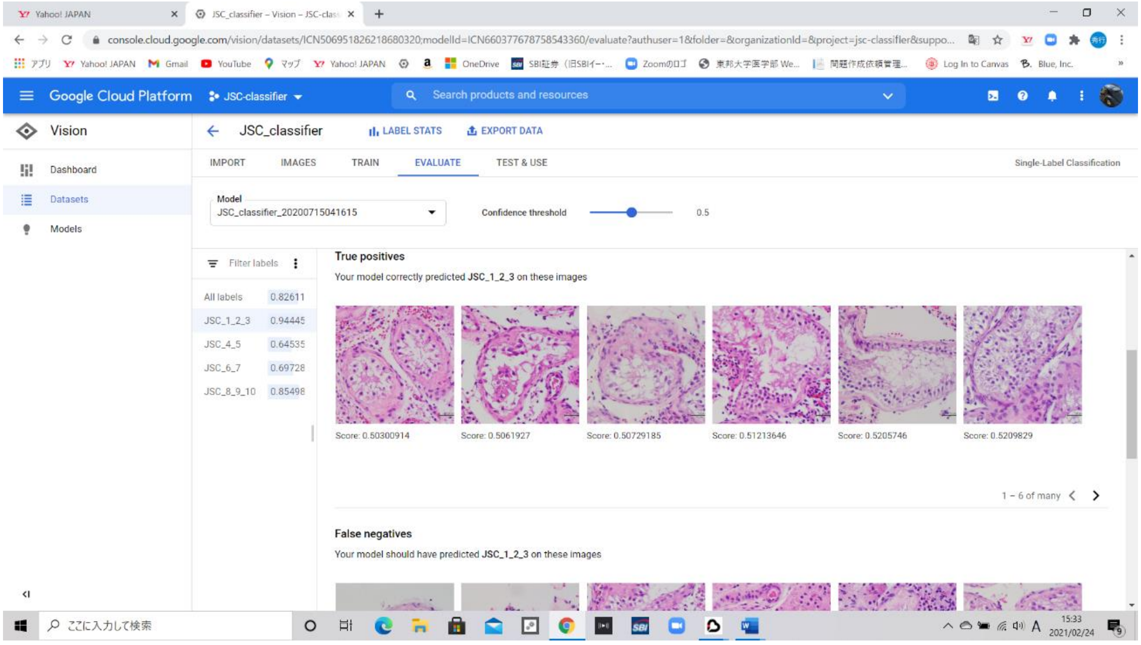

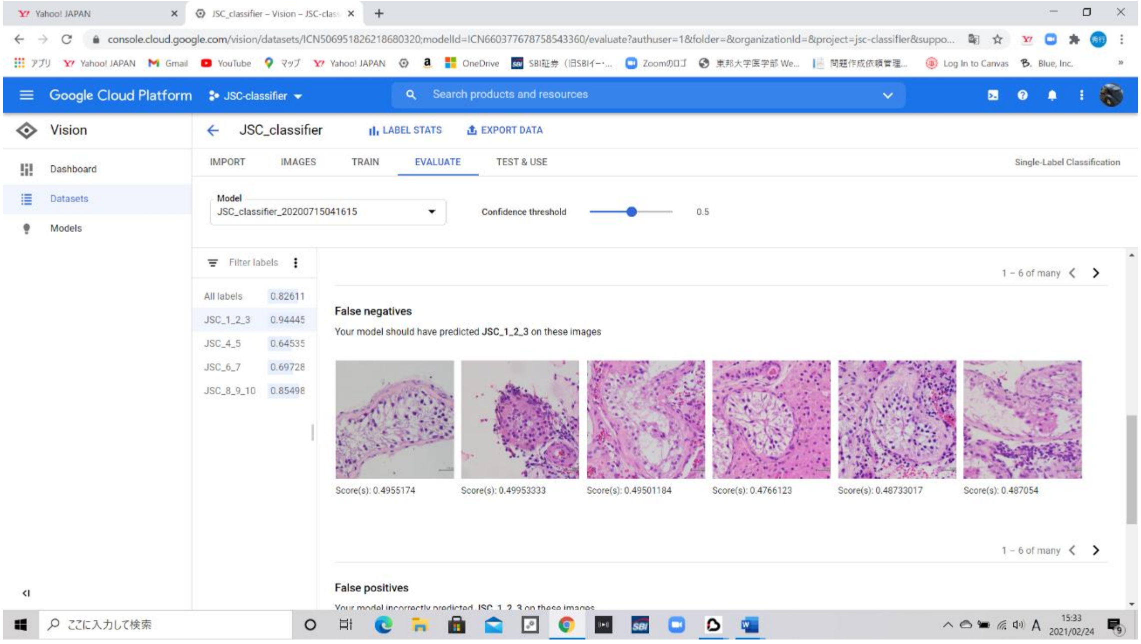
Google Cloud Platform
JSC-classifier
Search products and resources
Vision
JSC_classifier
LABEL STATS
EXPORT DATA
Dashboard
Datasets
Models
IMPORT
IMAGES
TRAIN
EVALUATE
TEST & USE
Single-Label Classification
Model
JSC_classifier_20200715041615
Confidence threshold
0.5
Filter labels
All labels 0.82611
JSC_1_2_3 0.94445
JSC_4_5 0.64535
JSC_6_7 0.69728
JSC_8_9_10 0.85498
1 – 6 of many
False negatives
Your model should have predicted JSC_1_2_3 on these images
Score(s): 0.4955174
Score(s): 0.49953333
Score(s): 0.49501184
Score(s): 0.4766123
Score(s): 0.48733017
Score(s): 0.487054
False positives
15:33
2021/02/24

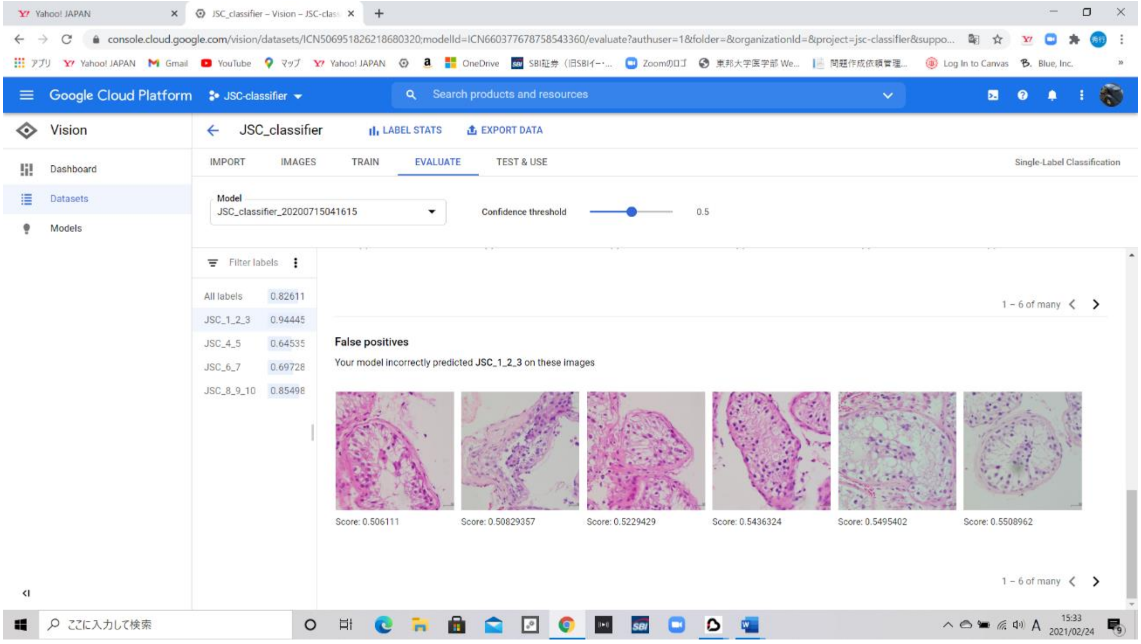
Google Cloud Platform
JSC-classifier
Search products and resources
Vision
JSC_classifier
LABEL STATS
EXPORT DATA
Dashboard
Datasets
Models
IMPORT
IMAGES
TRAIN
EVALUATE
TEST & USE
Single-Label Classification
Model
JSC_classifier_20200715041615
Confidence threshold
0.5
Filter labels
All labels 0.82611
JSC_1_2_3 0.94445
JSC_4_5 0.64535
JSC_6_7 0.69728
JSC_8_9_10 0.85498
1 – 6 of many
False positives
Your model incorrectly predicted JSC_1_2_3 on these images
Score: 0.506111
Score: 0.50829357
Score: 0.5229429
Score: 0.5436324
Score: 0.5495402
Score: 0.5508962
15:33
2021/02/24

The precision was 64.06% and recall was 37.77% for a Johnsen score of 4-5. True positive, false negative, and false positive images are indicated.

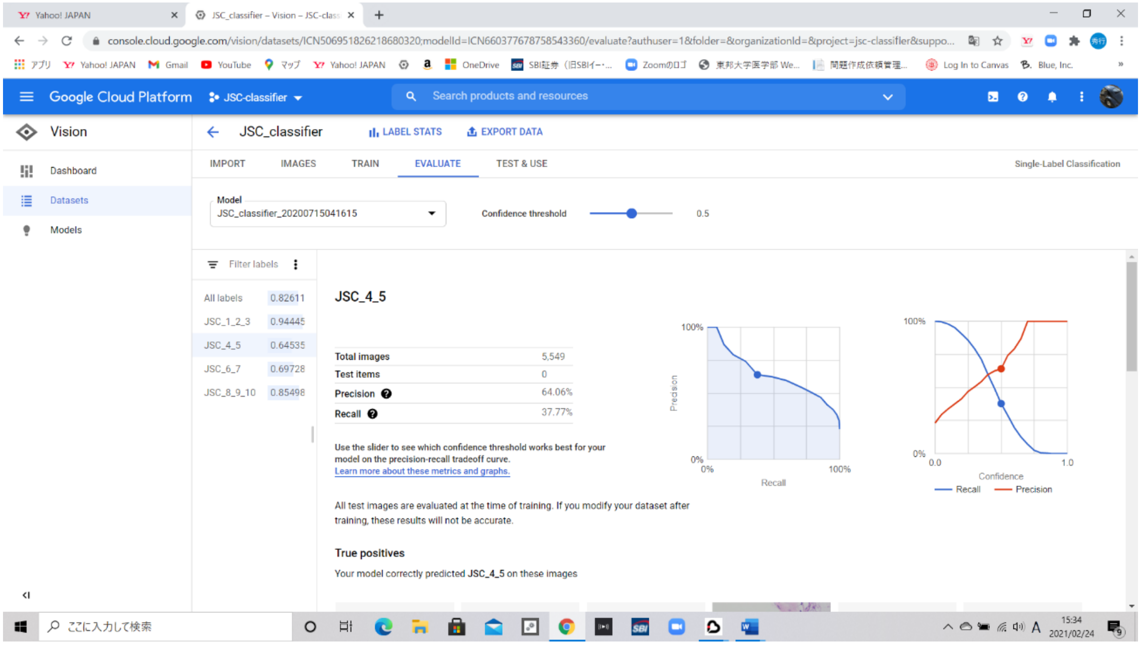

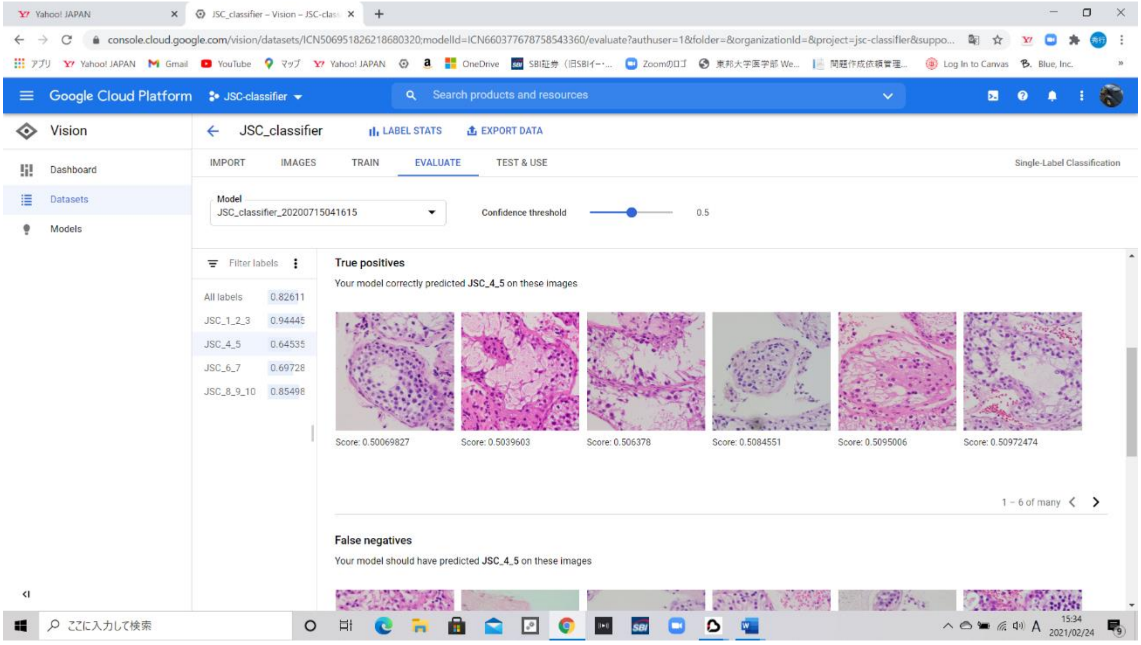

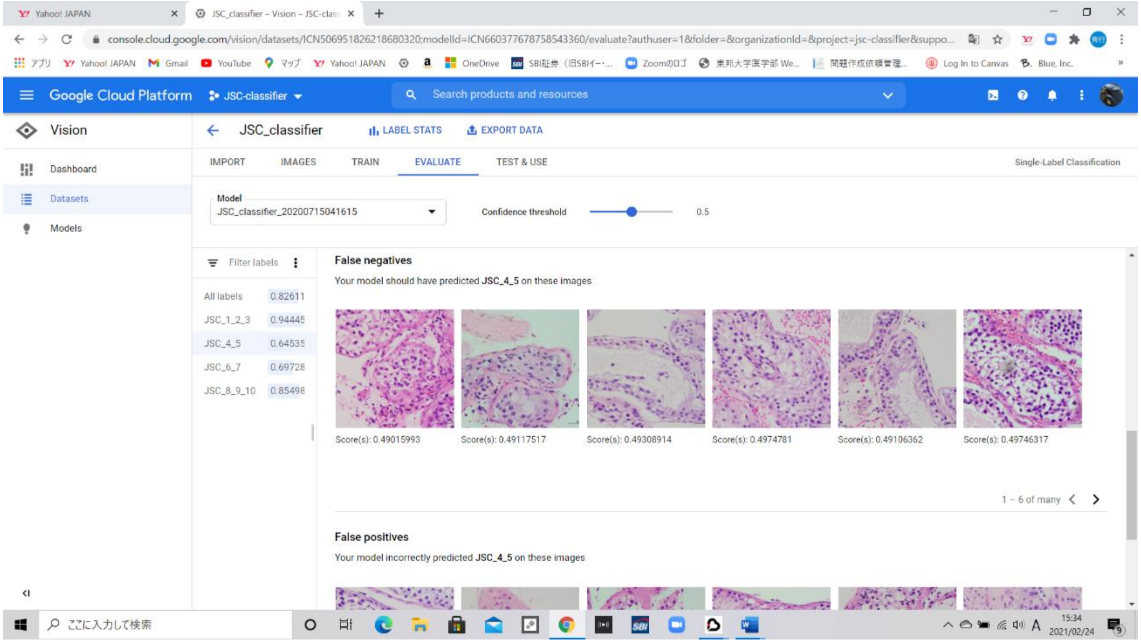

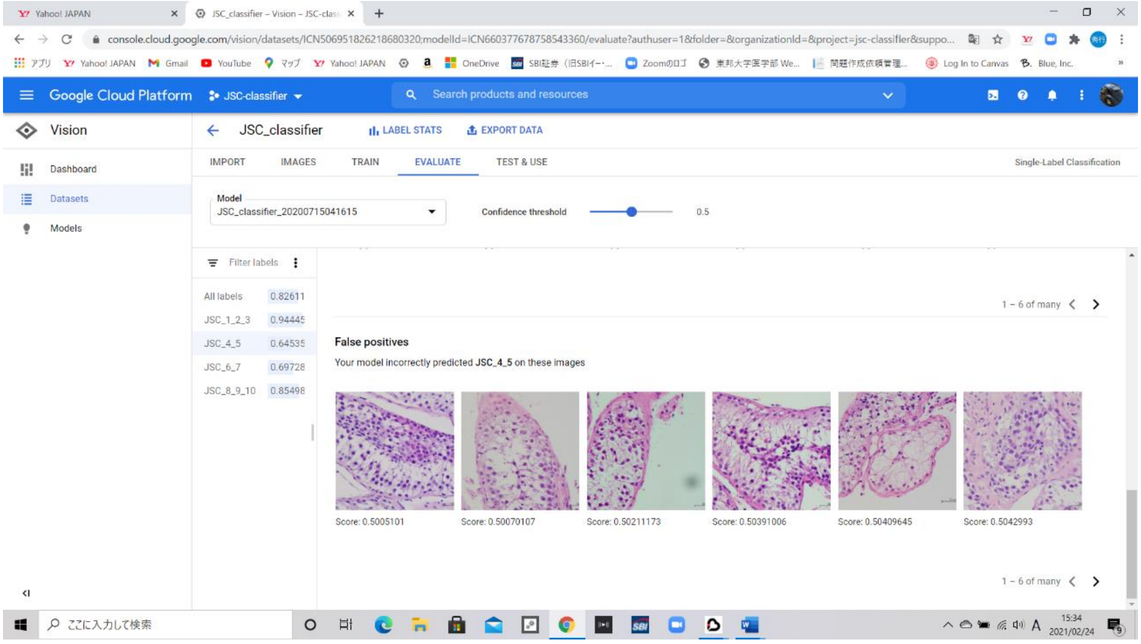

The precision was 72.39% and recall was 47.78% for a Johnsen score of 6-7. True positive, false negative, and false positive images are indicated.

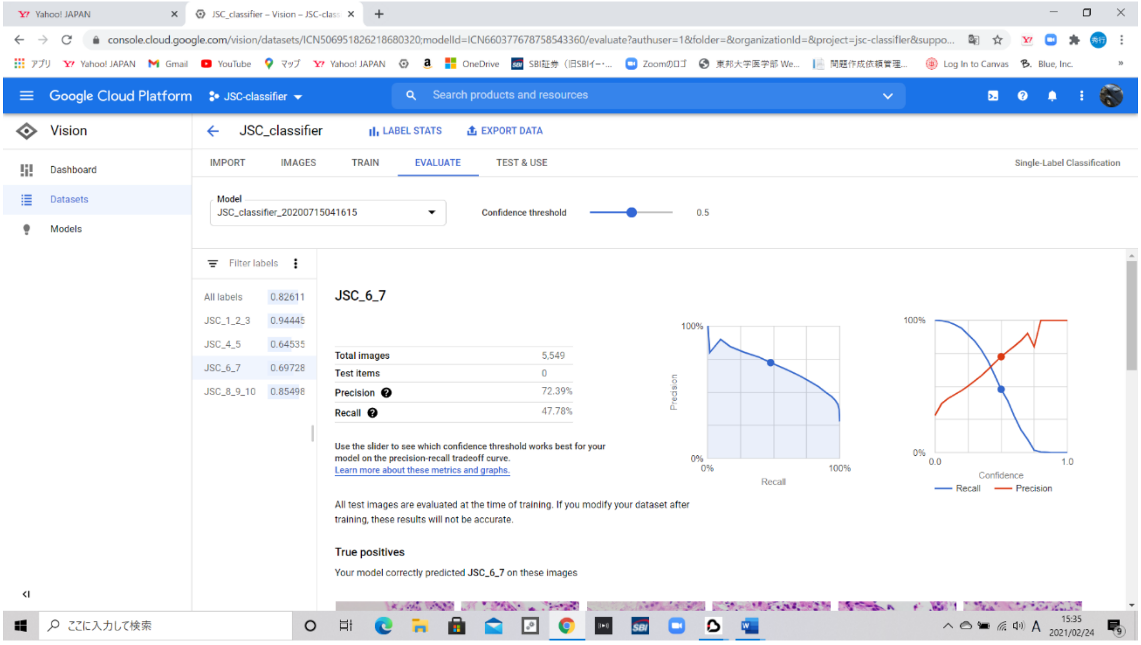

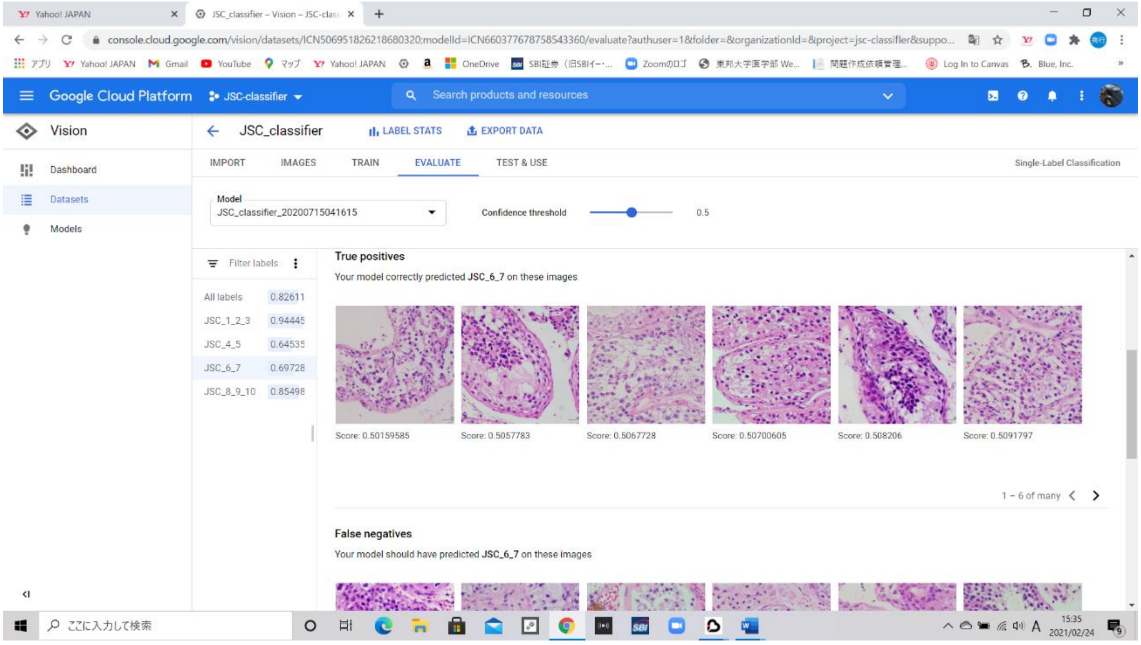

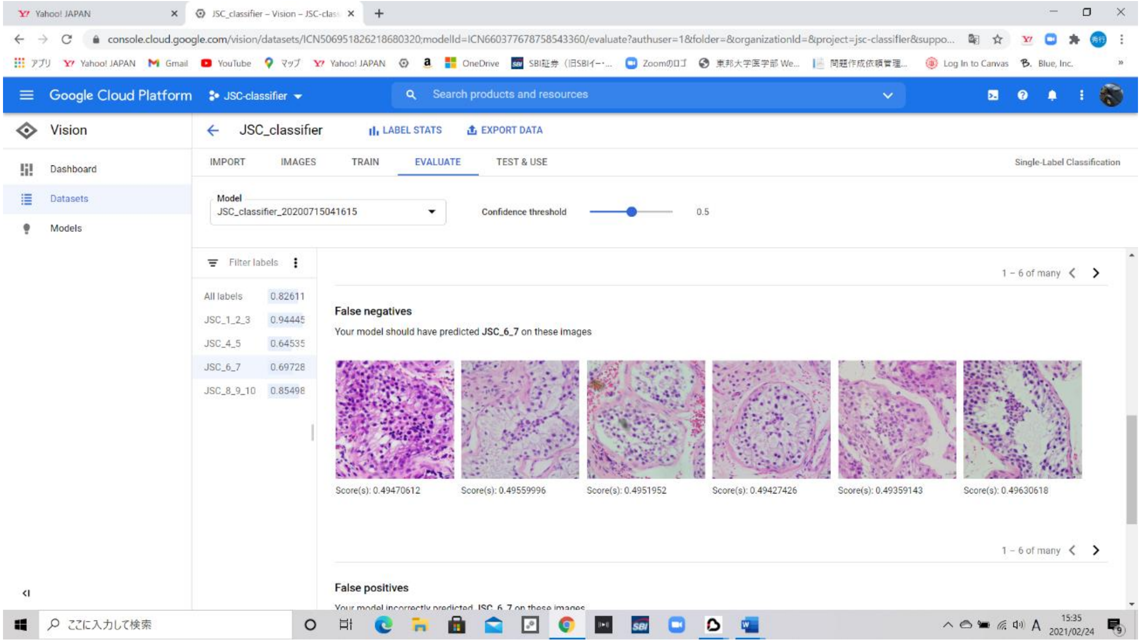

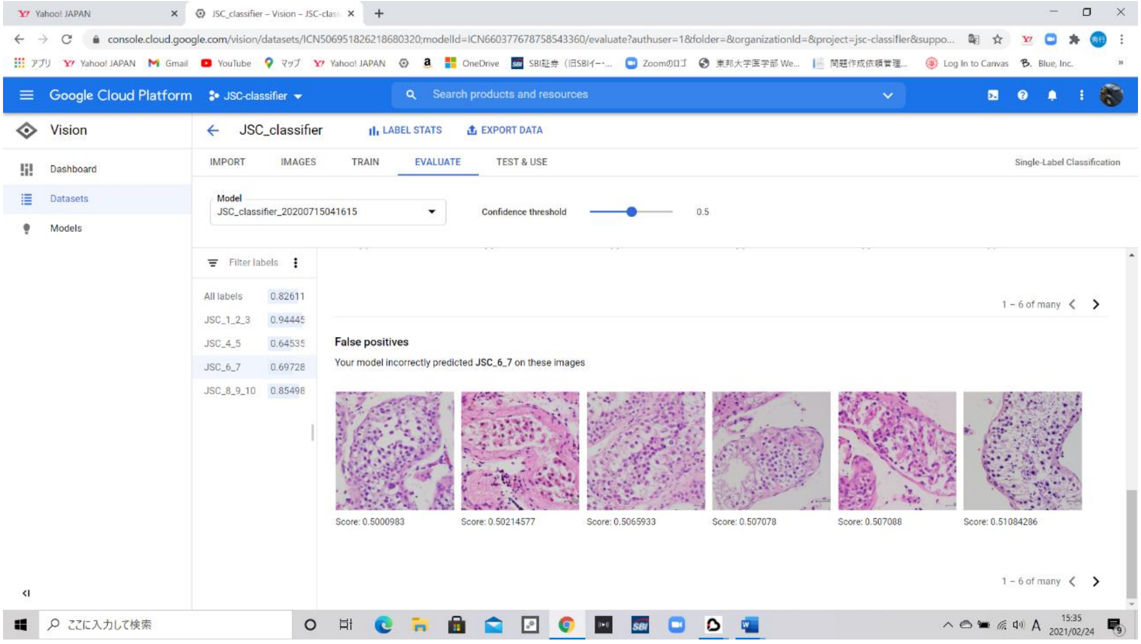

The precision was 88.37% and recall was 65.8% for a Johnsen score of 8-10. True positive, false negative, and false positive images are indicated.

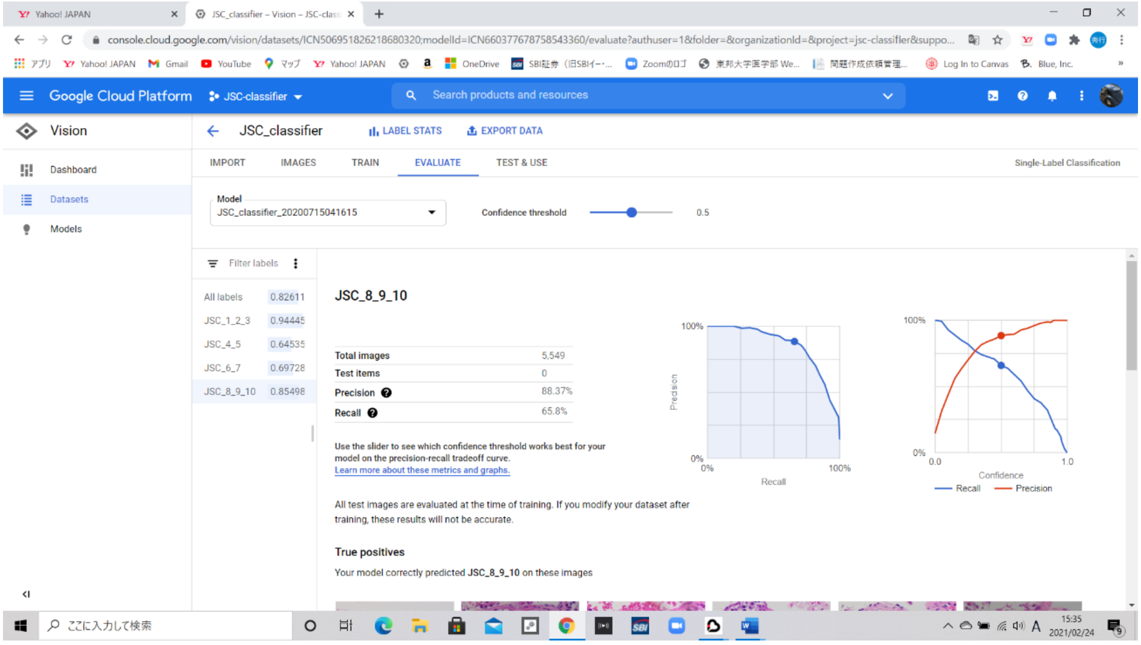

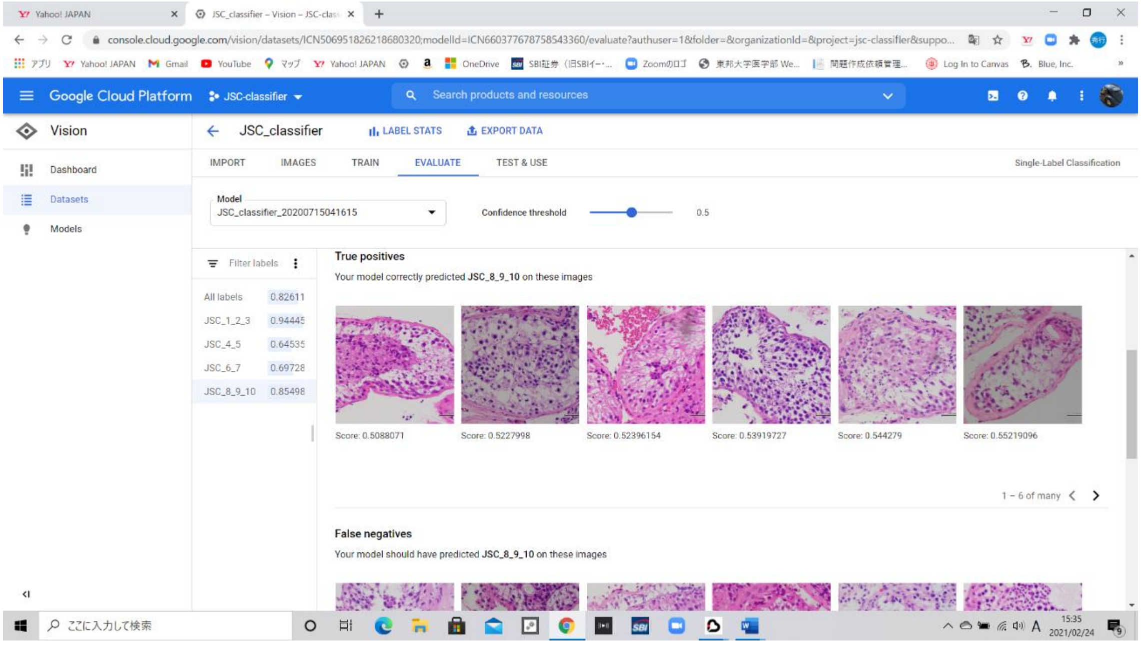

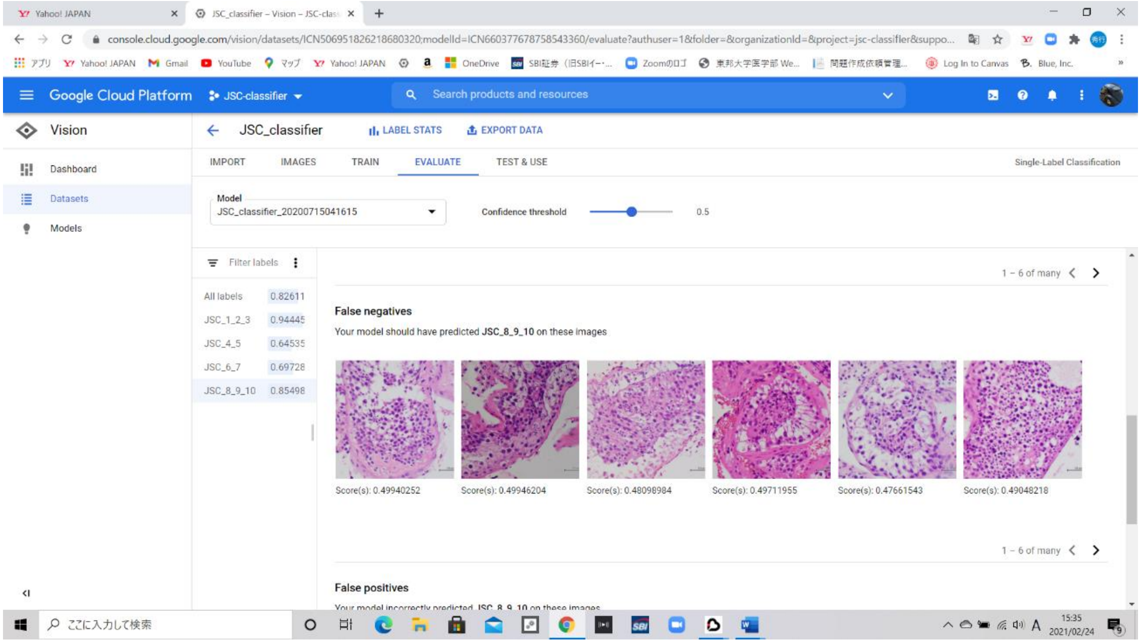

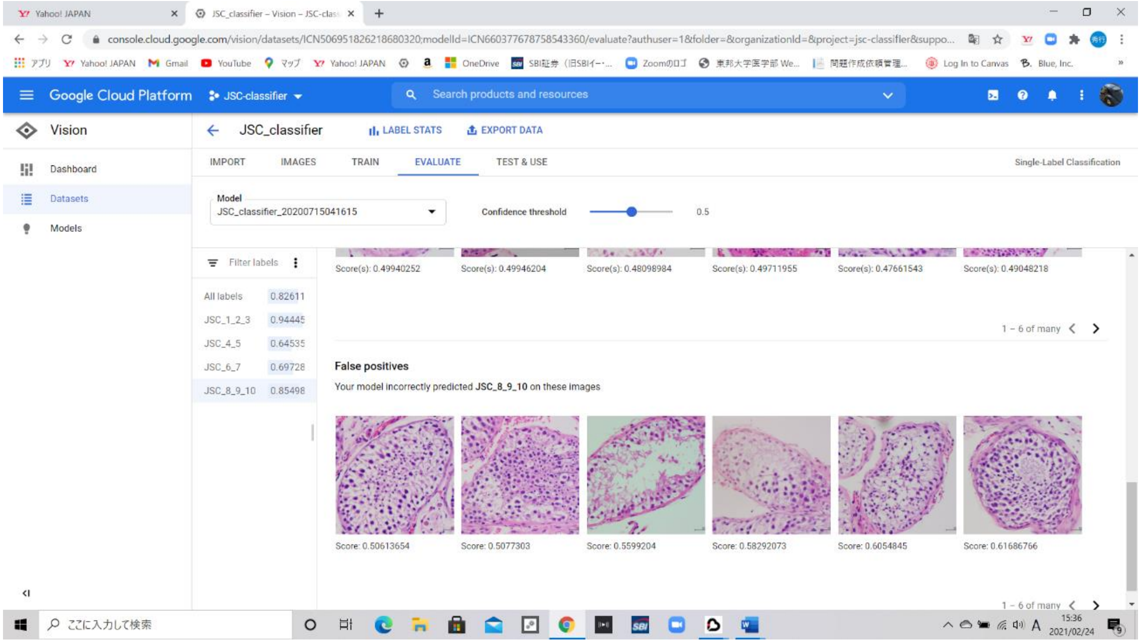

JSC_classifier_Part 2: image dataset of 9822 expansion images (5.0 X 5.0 cm)

The average precision (positive predictive value) of the algorithm was 99.5%, precision was 96.29%, and recall was 96.23% for all labels.

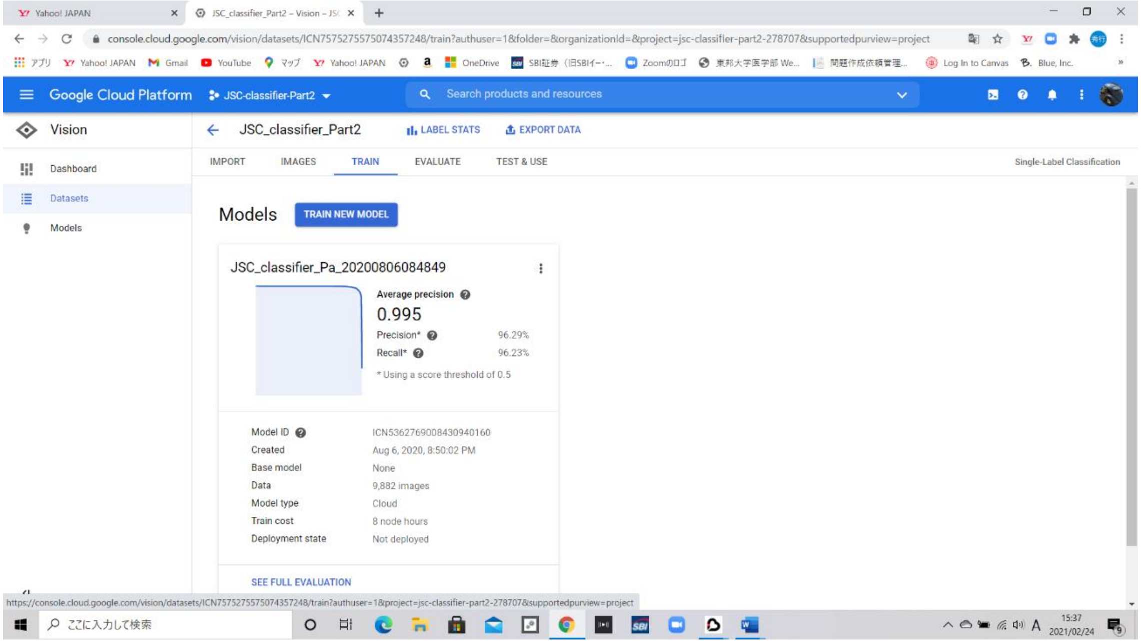

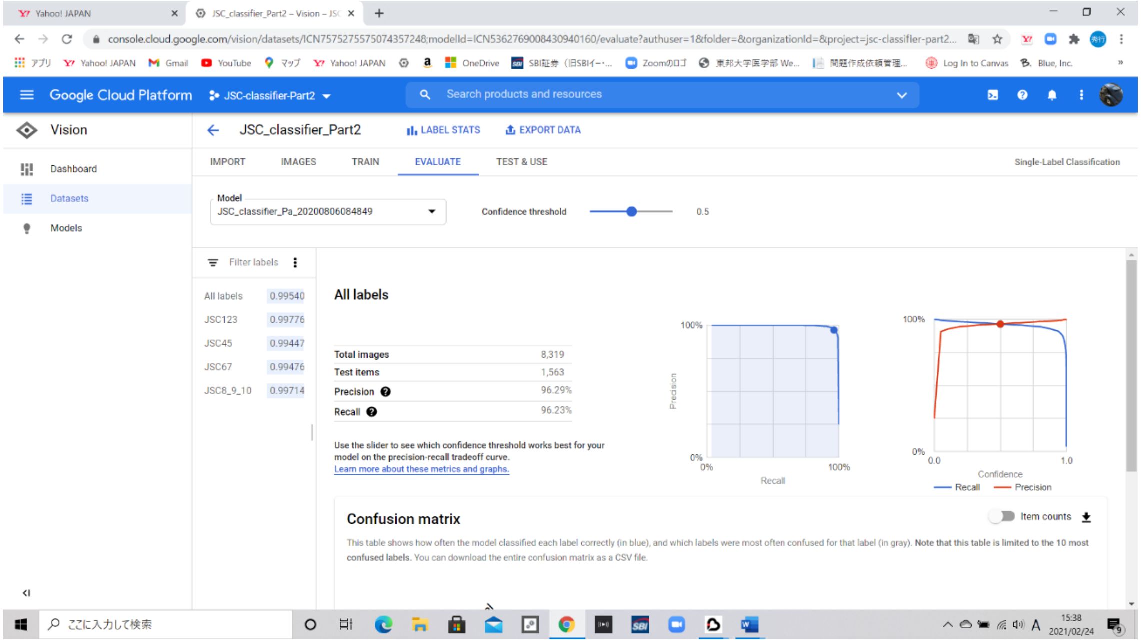

Confusion matrix

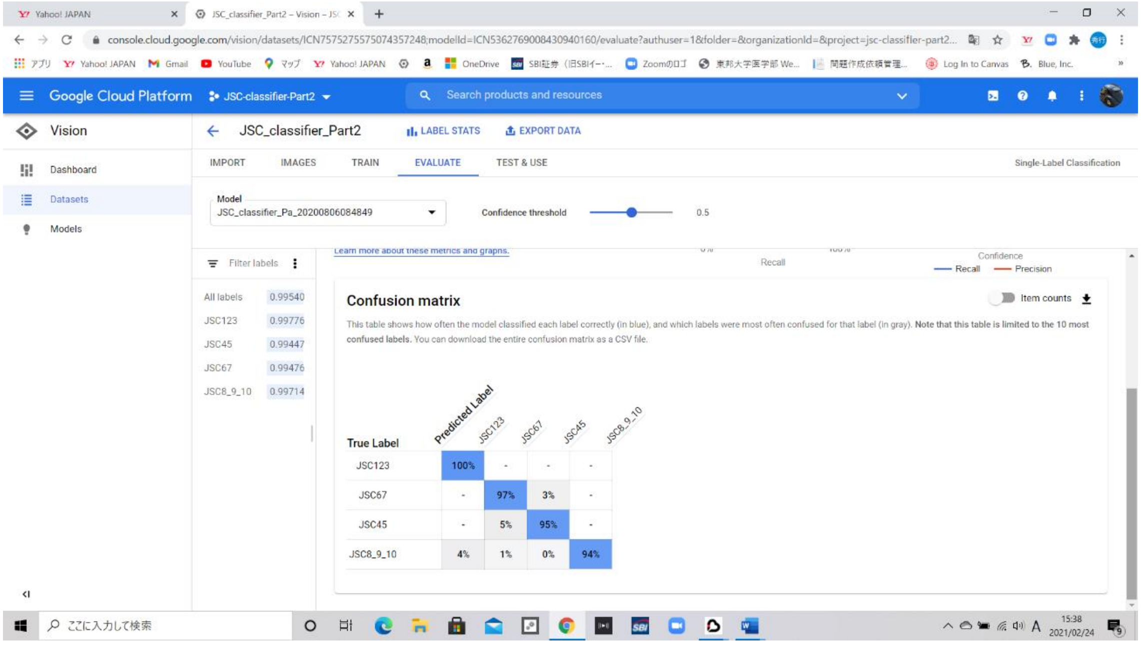

The precision was 95.82% and recall was 100% for a Johnsen score of 1-3. True positive and false positive images are indicated.

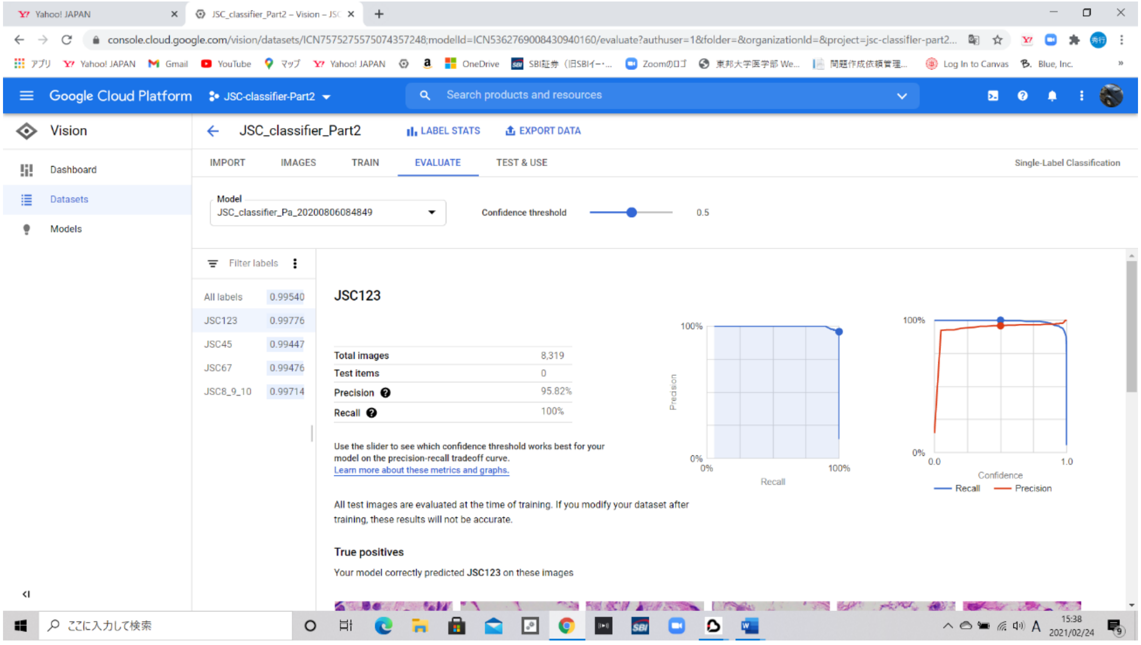

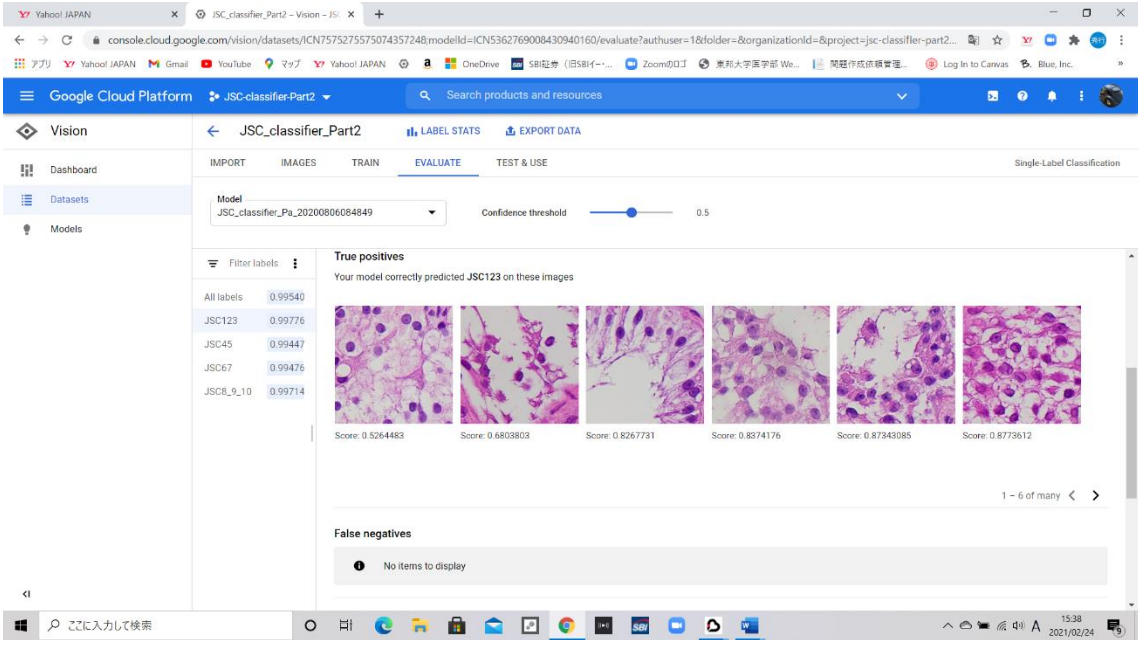

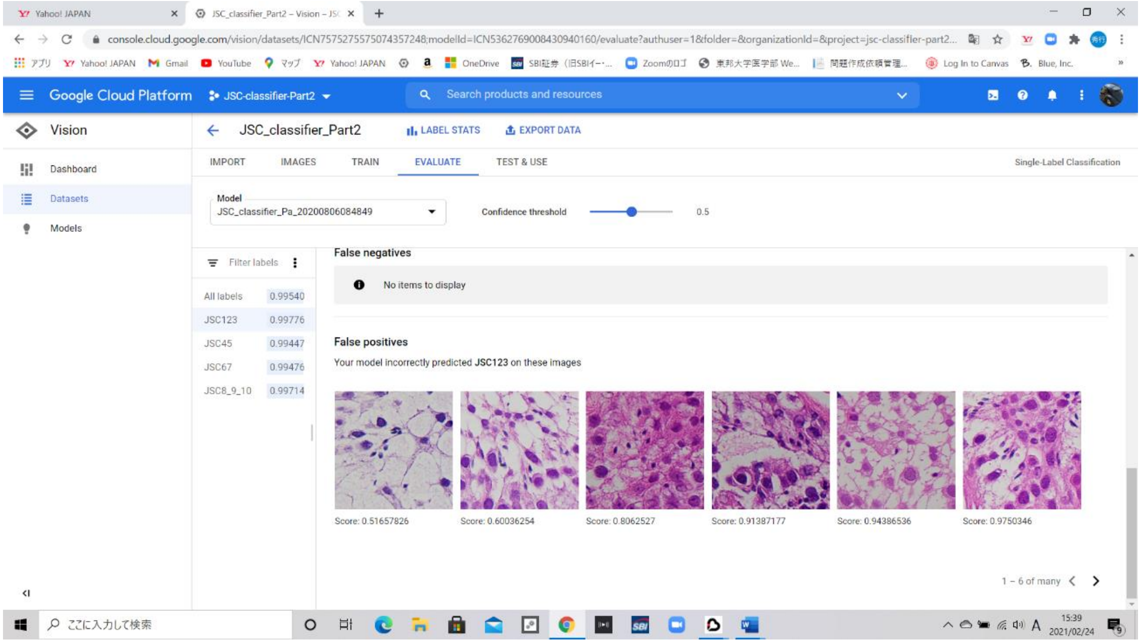
Google Cloud Platform
JSC-classifier-Part2
Search products and resources
Vision
Dashboard
Datasets
Models
JSC_classifier_Part2
LABEL STATS
EXPORT DATA
IMPORT
IMAGES
TRAIN
EVALUATE
TEST & USE
Single-Label Classification
Model
JSC_classifier_Pa_20200806084849
Confidence threshold
0.5
Filter labels
All labels 0.99540
JSC123 0.99776
JSC45 0.99447
JSC67 0.99476
JSC8_9_10 0.99714
False negatives
No items to display
False positives
Your model incorrectly predicted JSC123 on these images
Score: 0.51657826
Score: 0.60036254
Score: 0.8062527
Score: 0.91387177
Score: 0.94386536
Score: 0.9750346
1 – 6 of many

The precision was 95.7% and recall was 95.11% for a Johnsen score of 4-5. True positive, false negative, and false positive images are indicated.

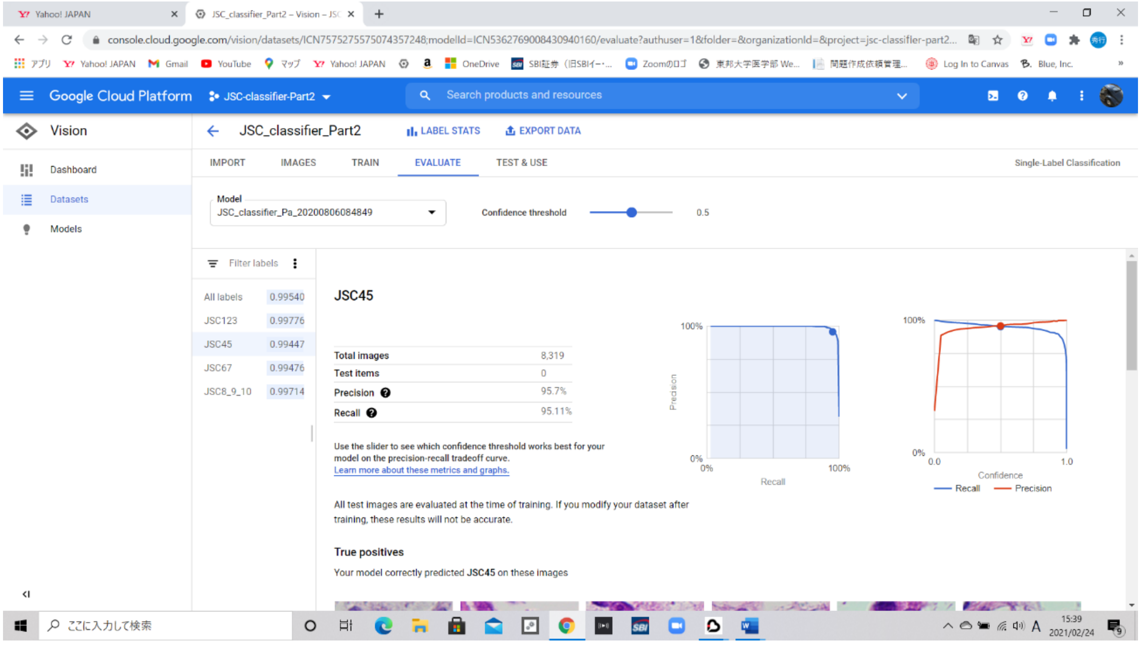

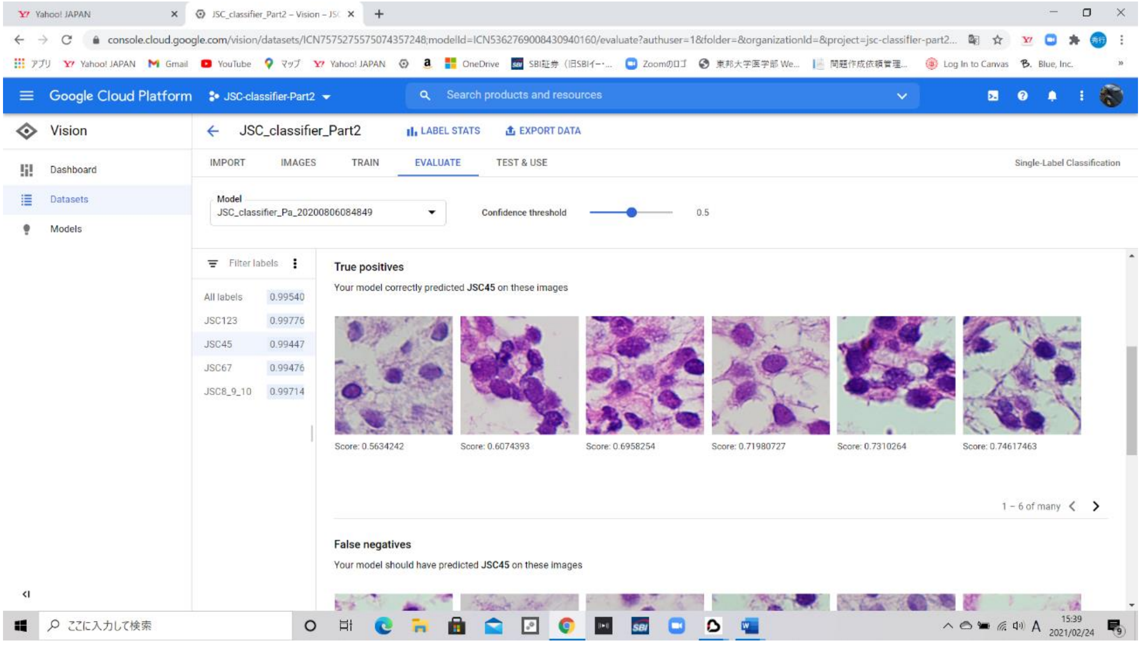

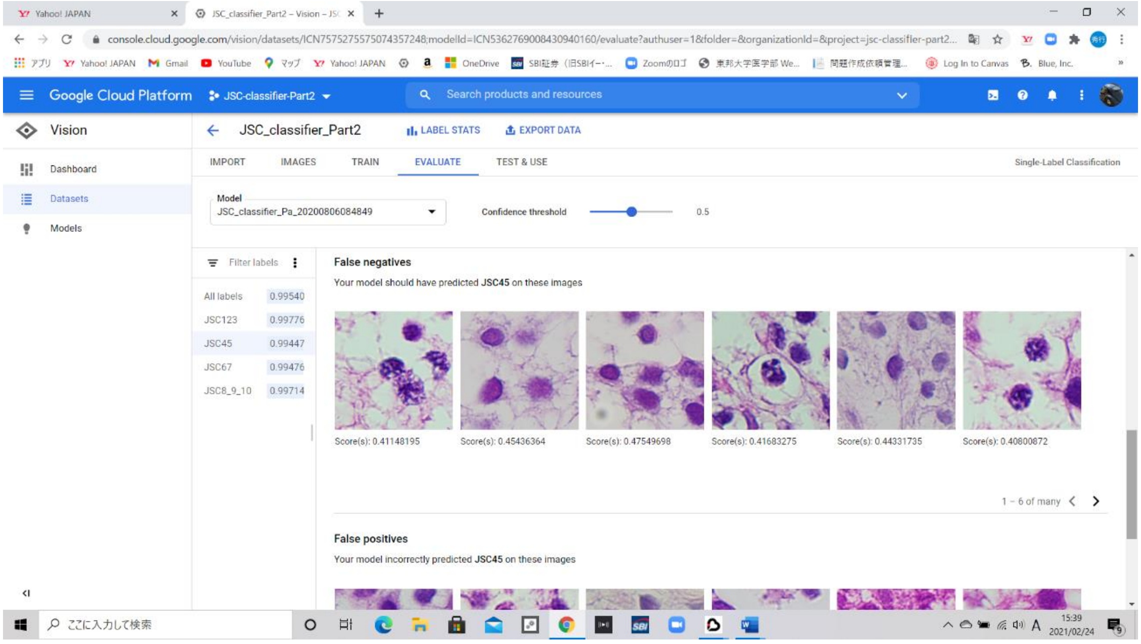
Yahoo! JAPAN
JSC_classifier_Part2 – Vision – JSC
console.cloud.google.com/vision/datasets/ICN7575275575074357248;modelId=ICN5362769008430940160/evaluate?authuser=1&folder=&organizationId=&project=jsc-classifier-part2...
Google Cloud Platform
JSC-classifier-Part2
Search products and resources
Vision
JSC_classifier_Part2
LABEL STATS
EXPORT DATA
Dashboard
Datasets
Models
IMPORT
IMAGES
TRAIN
EVALUATE
TEST & USE
Single-Label Classification
Model
JSC_classifier_Pa_20200806084849
Confidence threshold
0.5
Filter labels
All labels 0.99540
JSC123 0.99776
JSC45 0.99447
JSC67 0.99476
JSC8_9_10 0.99714
False negatives
Your model should have predicted JSC45 on these images
Score(s): 0.41148195
Score(s): 0.45436364
Score(s): 0.47549698
Score(s): 0.41683275
Score(s): 0.44331735
Score(s): 0.40800872
1 – 6 of many
False positives
Your model incorrectly predicted JSC45 on these images
ここに入力して検索
15:39
2021/02/24

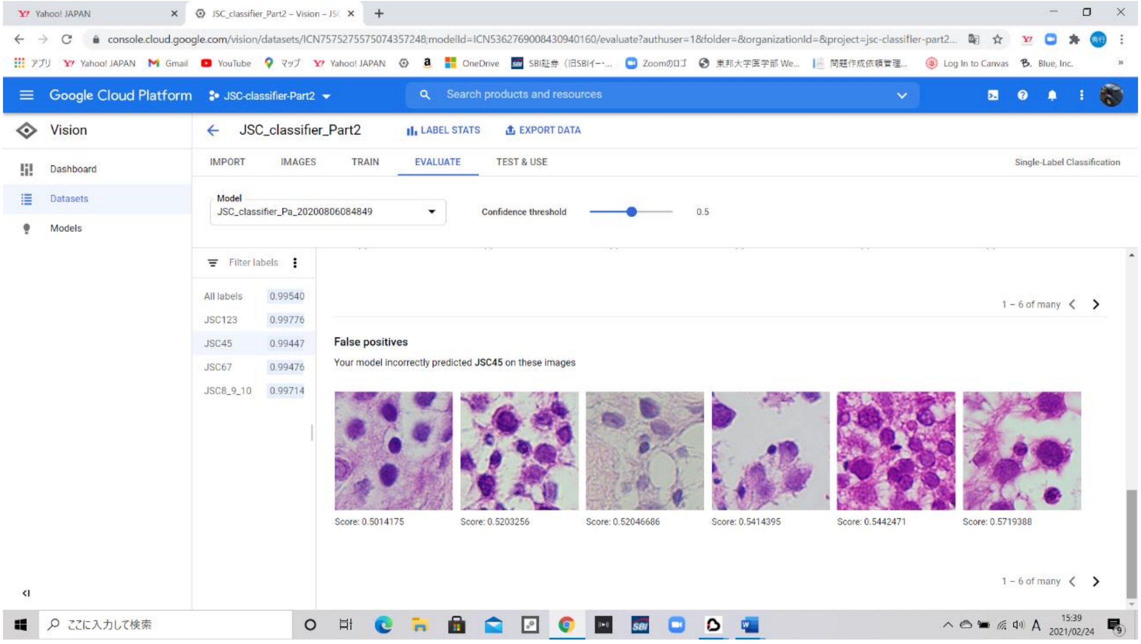
Yahoo! JAPAN
JSC_classifier_Part2 – Vision – JSC
console.cloud.google.com/vision/datasets/ICN7575275575074357248;modelId=ICN5362769008430940160/evaluate?authuser=1&folder=&organizationId=&project=jsc-classifier-part2...
Google Cloud Platform
JSC-classifier-Part2
Search products and resources
Vision
JSC_classifier_Part2
LABEL STATS
EXPORT DATA
Dashboard
Datasets
Models
IMPORT
IMAGES
TRAIN
EVALUATE
TEST & USE
Single-Label Classification
Model
JSC_classifier_Pa_20200806084849
Confidence threshold
0.5
Filter labels
All labels 0.99540
JSC123 0.99776
JSC45 0.99447
JSC67 0.99476
JSC8_9_10 0.99714
1 – 6 of many
False positives
Your model incorrectly predicted JSC45 on these images
Score: 0.5014175
Score: 0.5203256
Score: 0.52046686
Score: 0.5414395
Score: 0.5442471
Score: 0.5719388
1 – 6 of many
ここに入力して検索
15:39
2021/02/24

The precision was 95.51% and recall was 96.47% for a Johnsen score of 6-7. True positive, false negative, and false positive images are indicated.

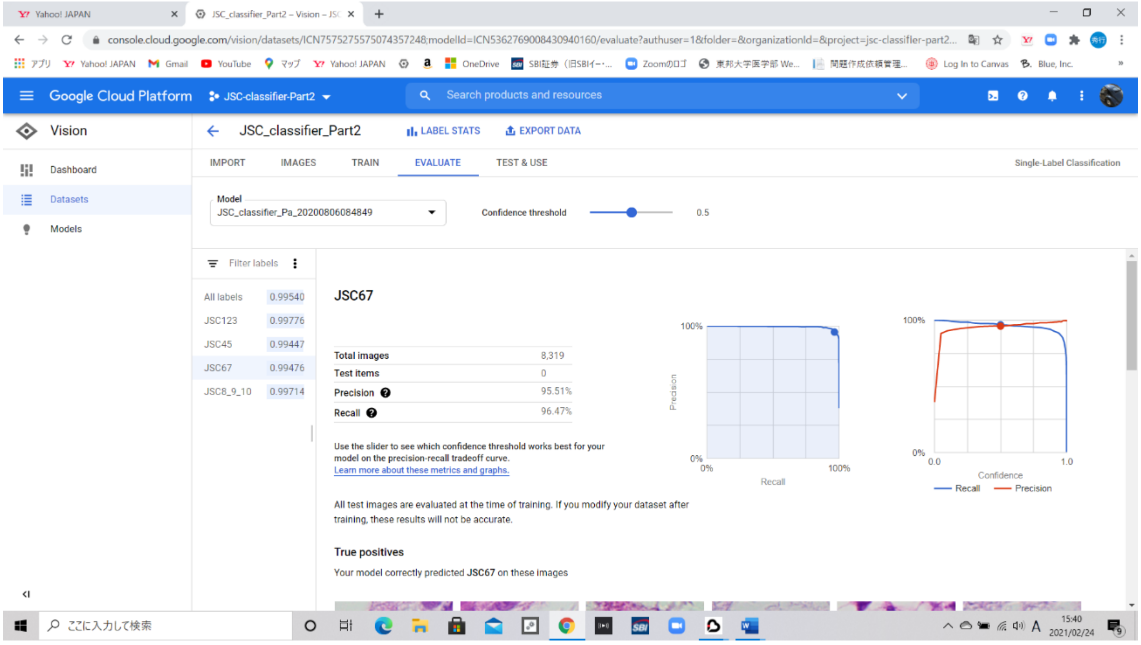

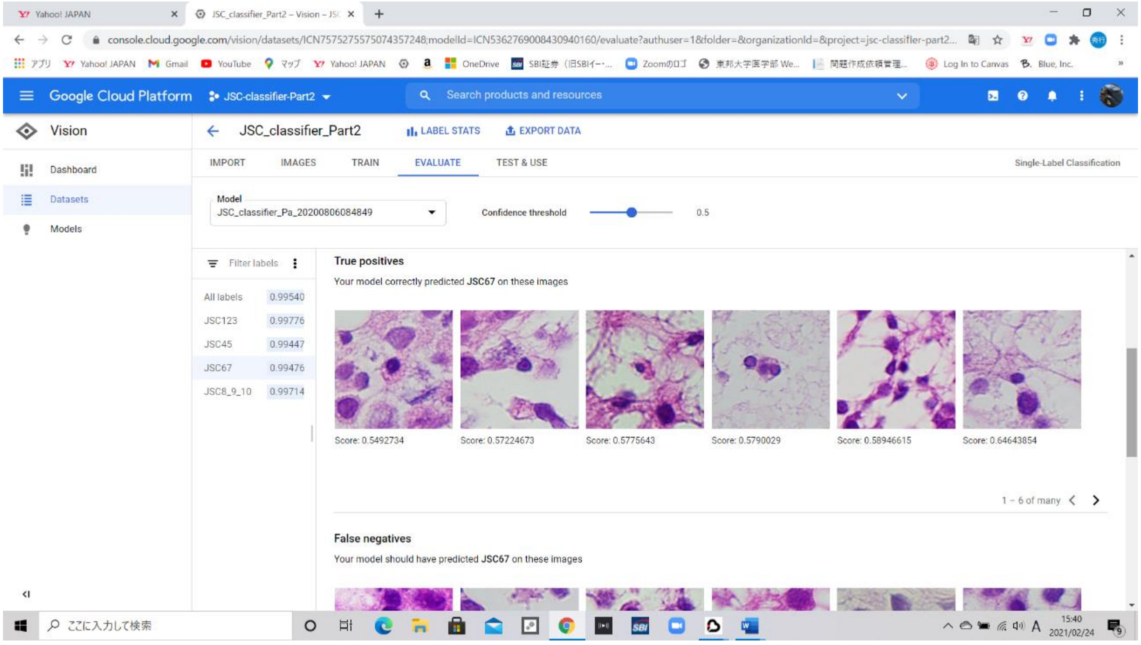

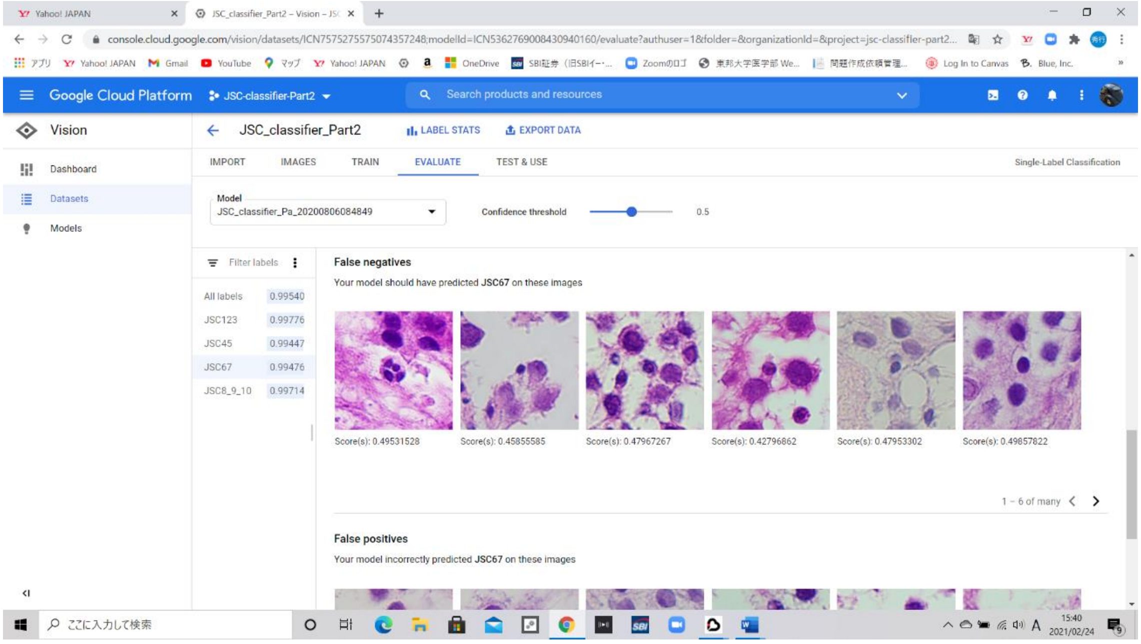
Google Cloud Platform
JSC-classifier-Part2
Search products and resources
Vision
JSC_classifier_Part2
LABEL STATS
EXPORT DATA
IMPORT
IMAGES
TRAIN
EVALUATE
TEST & USE
Single-Label Classification
Dashboard
Datasets
Models
Model
JSC_classifier_Pa_20200806084849
Confidence threshold
0.5
Filter labels
All labels 0.99540
JSC123 0.99776
JSC45 0.99447
JSC67 0.99476
JSC8_9_10 0.99714
False negatives
Your model should have predicted JSC67 on these images
Score(s): 0.49531528
Score(s): 0.45855585
Score(s): 0.47967267
Score(s): 0.42796862
Score(s): 0.47953302
Score(s): 0.49857822
1 – 6 of many
False positives
Your model incorrectly predicted JSC67 on these images
ここに入力して検索
15:40
2021/02/24

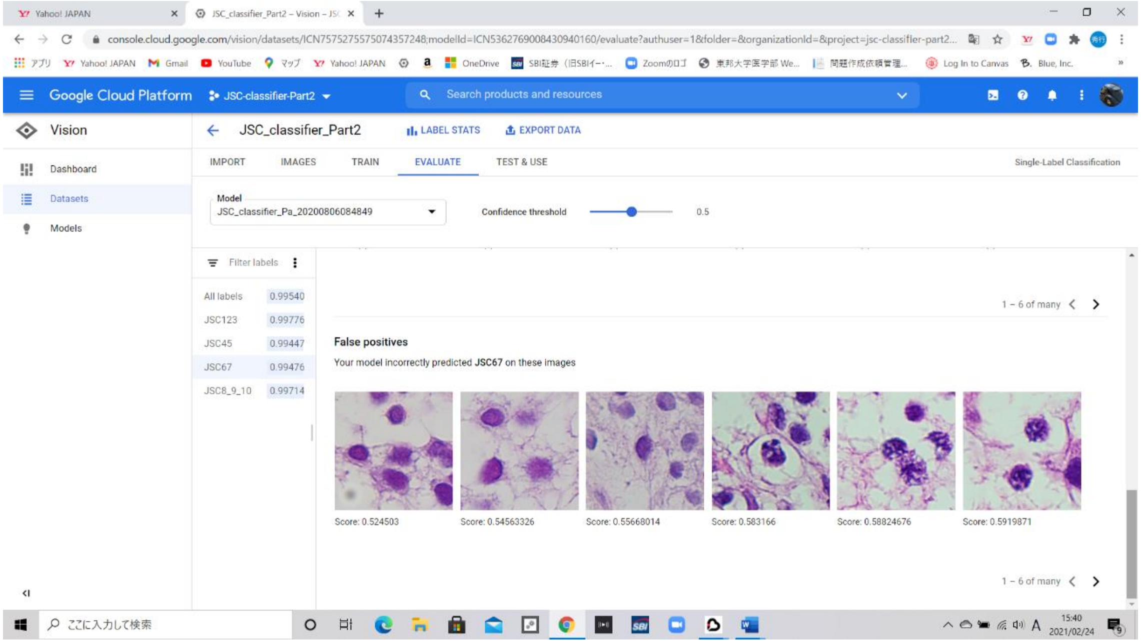
Google Cloud Platform
JSC-classifier-Part2
Search products and resources
Vision
JSC_classifier_Part2
LABEL STATS
EXPORT DATA
IMPORT
IMAGES
TRAIN
EVALUATE
TEST & USE
Single-Label Classification
Dashboard
Datasets
Models
Model
JSC_classifier_Pa_20200806084849
Confidence threshold
0.5
Filter labels
All labels 0.99540
JSC123 0.99776
JSC45 0.99447
JSC67 0.99476
JSC8_9_10 0.99714
1 – 6 of many
False positives
Your model incorrectly predicted JSC67 on these images
Score: 0.524503
Score: 0.54563326
Score: 0.55668014
Score: 0.583166
Score: 0.58824676
Score: 0.5919871
1 – 6 of many
ここに入力して検索
15:40
2021/02/24

The precision was 100% and recall was 94.35% for a Johnsen score of 8-10. True positive and false negative images are indicated.

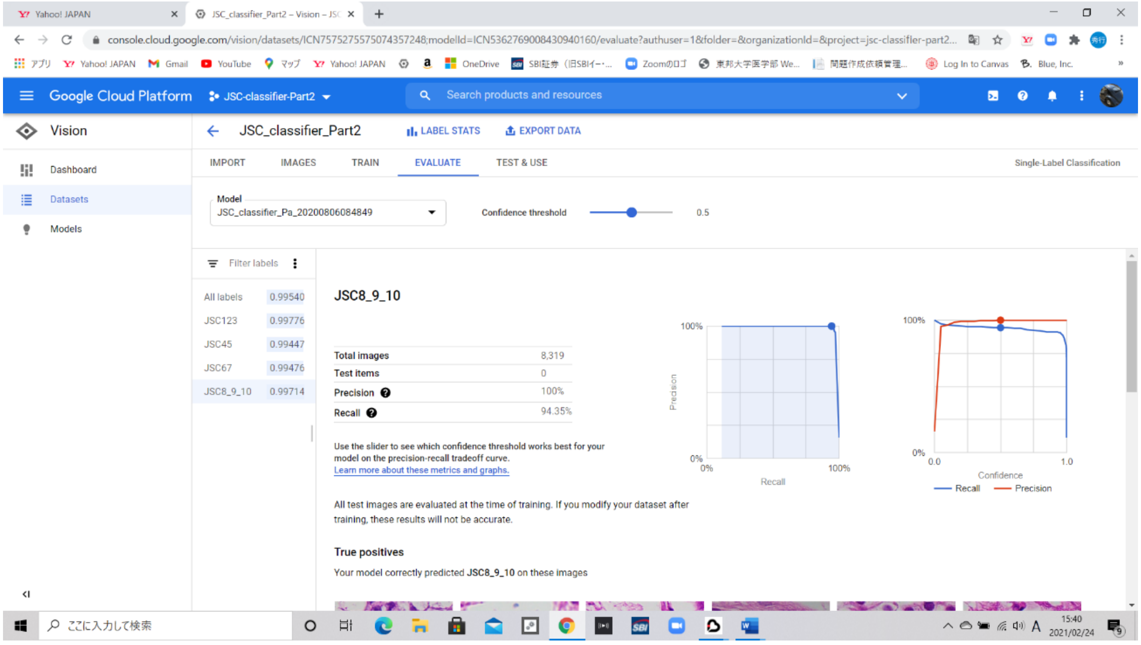

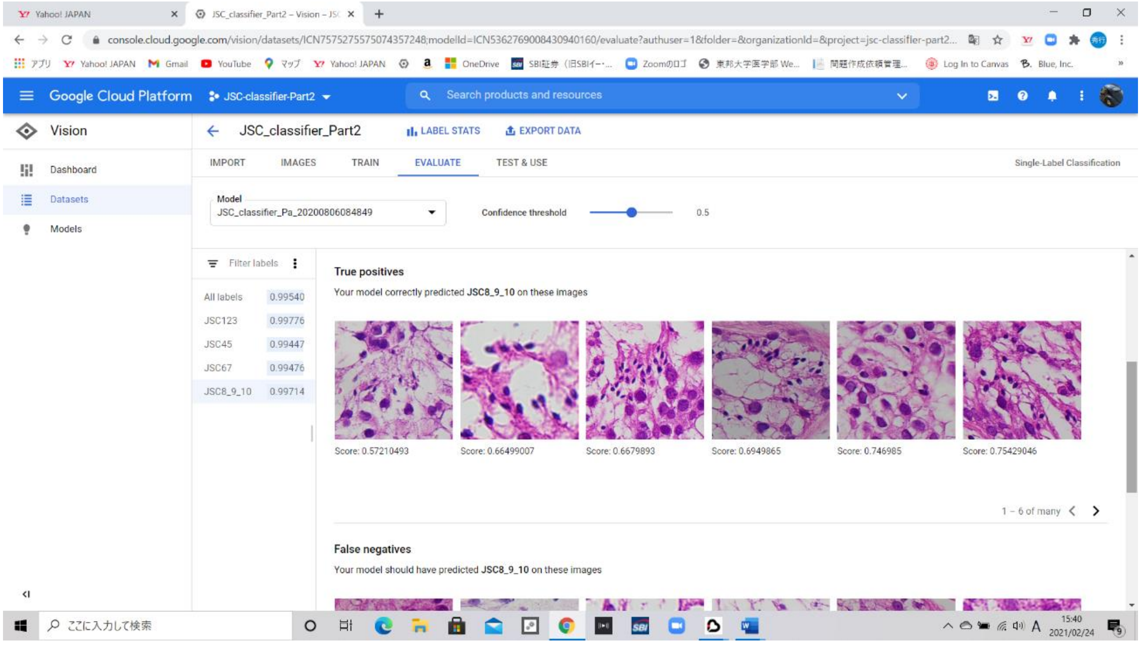

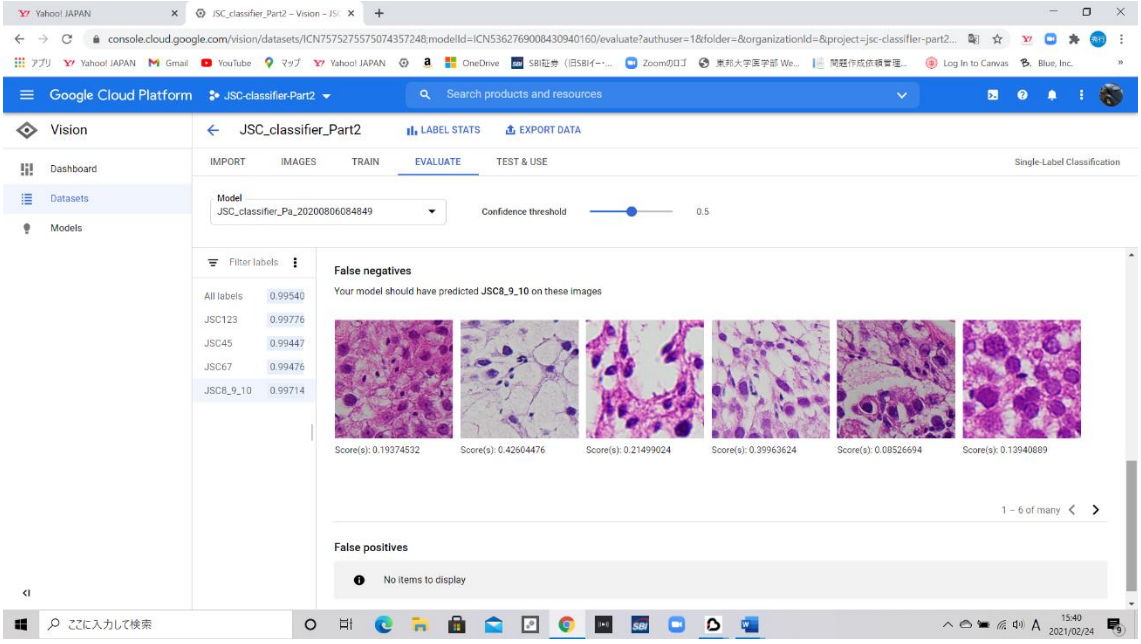
Google Cloud Platform
JSC-classifier-Part2
Search products and resources
Vision
JSC_classifier_Part2
LABEL STATS
EXPORT DATA
IMPORT
IMAGES
TRAIN
EVALUATE
TEST & USE
Single-Label Classification
Dashboard
Datasets
Models
Model
JSC_classifier_Pa_20200806084849
Confidence threshold
0.5
Filter labels
All labels 0.99540
JSC123 0.99776
JSC45 0.99447
JSC67 0.99476
JSC8_9_10 0.99714
False negatives
Your model should have predicted JSC8_9_10 on these images
Score(s): 0.19374532
Score(s): 0.42604476
Score(s): 0.21499024
Score(s): 0.39963624
Score(s): 0.08526694
Score(s): 0.13940889
1 – 6 of many
False positives
No items to display
ここに入力して検索
15:40
2021/02/24
