# Supplementary material for: A method for utilizing automated machine learning for histopathological classification of testis based on Johnsen scores
Source: Sci Rep. 2021 May 10;11:9962. doi: 10.1038/s41598-021-89369-z (PMC8107178; doi:10.1038/s41598-021-89369-z)

A method for utilizing automated machine learning for histopathological classification of testis based on Johnsen scores

Yurika Ito[1], Mami Unagami[1], Fumito Yamabe[1], Yozo Mitsui[1], Koichi Nakajima[1], Koichi Nagao[1], Hideyuki Kobayashi[1,*]

[1] Department of Urology, Toho University School of Medicine, Tokyo, Japan

[*]To whom correspondence should be addressed: Department of Urology, Toho University School of Medicine, Tokyo, Japan, 6-11-1, Omori-Nishi, Ota-ku, Tokyo 143-8541, Japan

TEL +81-3-3762-4151 FAX +81-3-3768-8817

E-mail: hideyukk@med.toho-u.ac.jp

Supplementary information:Methods

How to use the image recognition model utilizing Google Cloud AutoML Vision.

Cloud AutoML Vision is a service provided on Google Cloud Platform. AutoML Vision makes it easy to create a deep learning model using prepared images without any knowledge of deep learning.

This is explained in detail at the following URL:. https://cloud.google.com/vision

Below, we explain the process of using Google Cloud AutoML Vision with our image dataset of 7155 magnified images (X400).

1. First, register an account on Google Cloud Platform (GCP). This is the home screen of the GCP console.

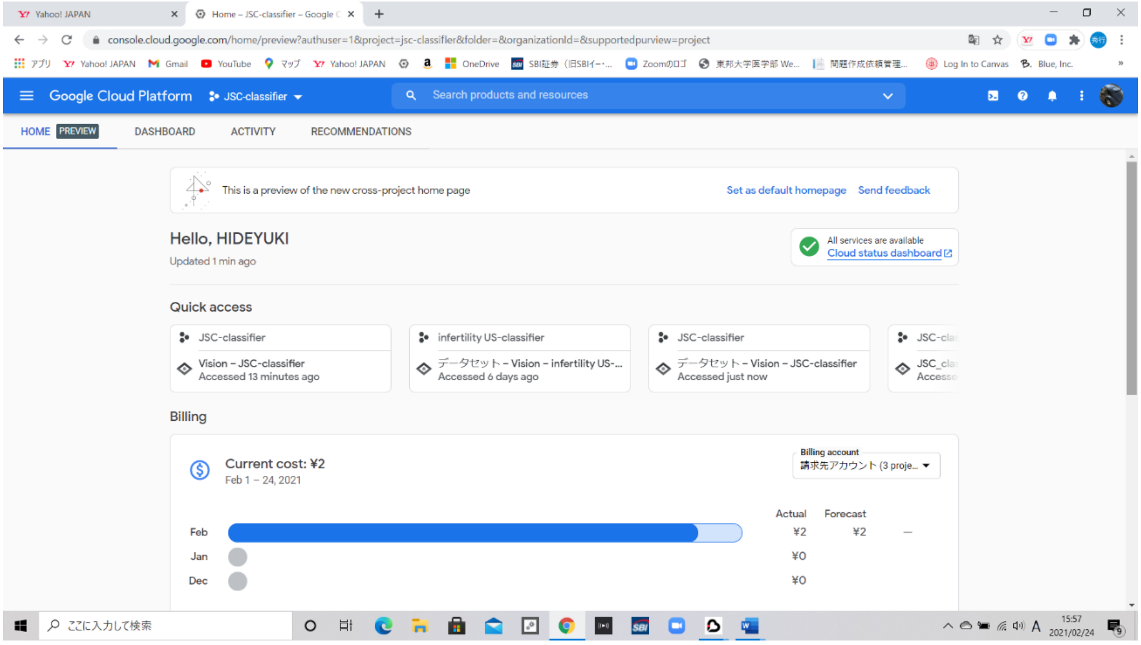

2. Next, make the settings for Cloud AutoML Vision on the GCP console to set up a GCP Project. This is the screen for “Select a project”. Click “NEW PROJECT” to initiate a GCP Project and decide on a project name. “NEW PROJECT” is enclosed in a red frame.

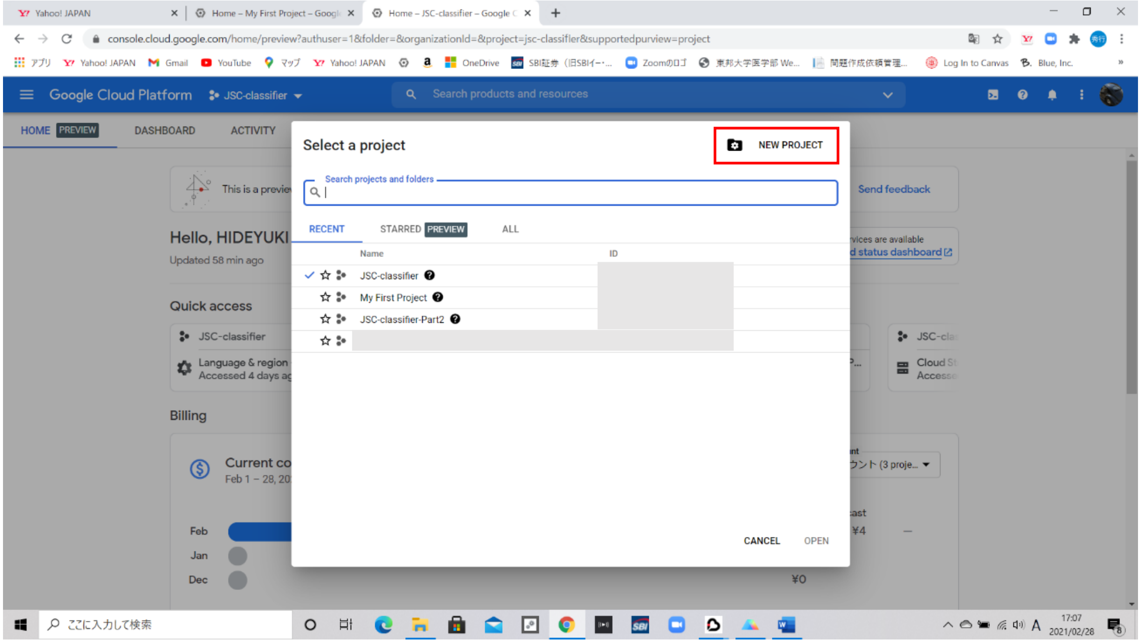

3. Then, activate Cloud AutoML Vision on the GCP console. Click “Vision” on the GCP console and carry out the process for activation of Cloud AutoML Vision. In addition, select “AutoML Vision” and “Get started” enclosed in a red frame.

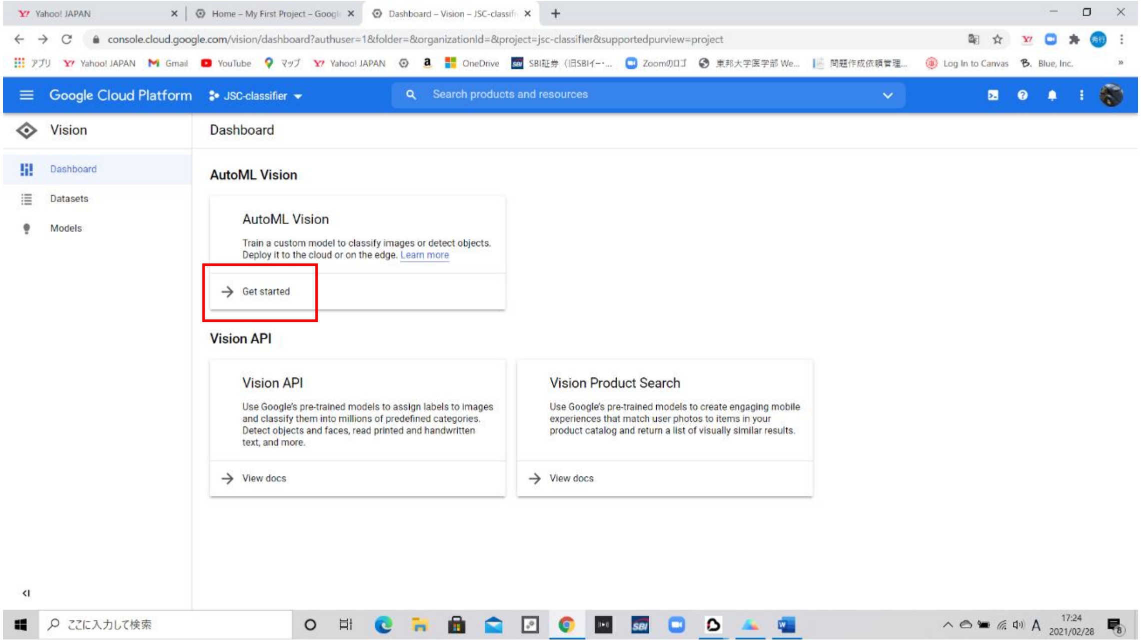

4. The next step is creating a Cloud Storage bucket. Click “Storage” on the GCP console. The bucket is like a box for saving images. In some cases, the bucket may be created automatically by activating a Cloud AutoML Vision project. If a bucket has not been created, carry out the process to create one manually. On the screen you see after clicking “Storage”, click “CREATE BUCKET”, which is enclosed in a red frame.

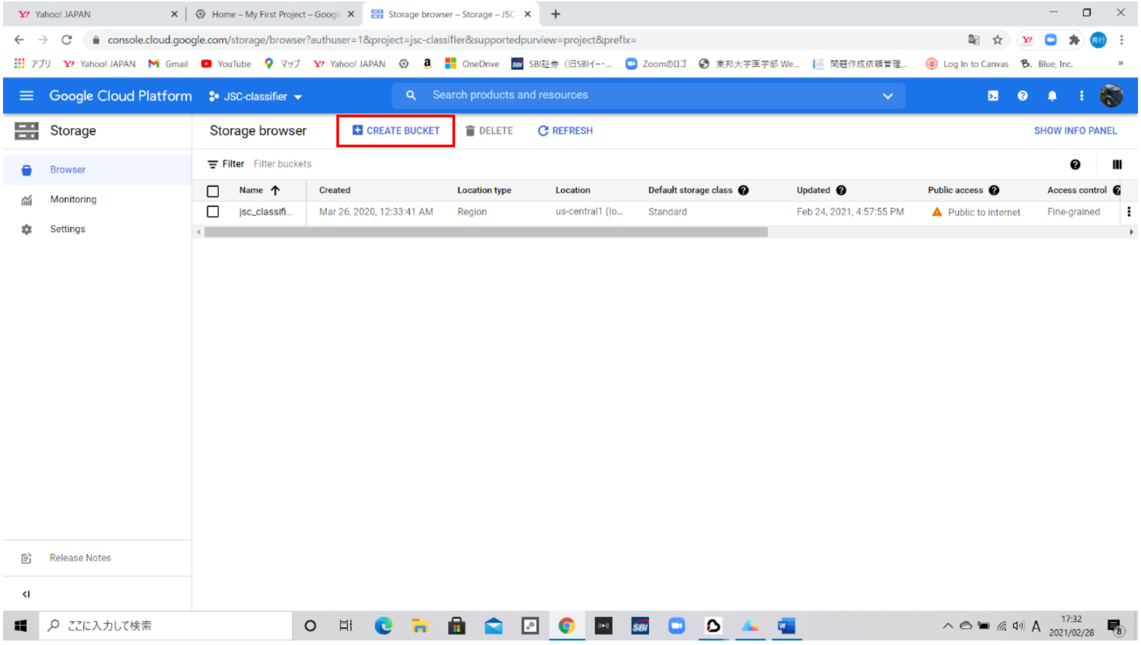

5. Then decide the “Name”, “Location type”, “Location”, and “Storage class”. The recommended format for the name is: {project ID}-vcm. Select “Region” as the Location type, “us-central1 (Iowa) as the Location, and “Standard” as the storage class. “Region”, “Location”, and “Storage class” are enclosed in red frames.

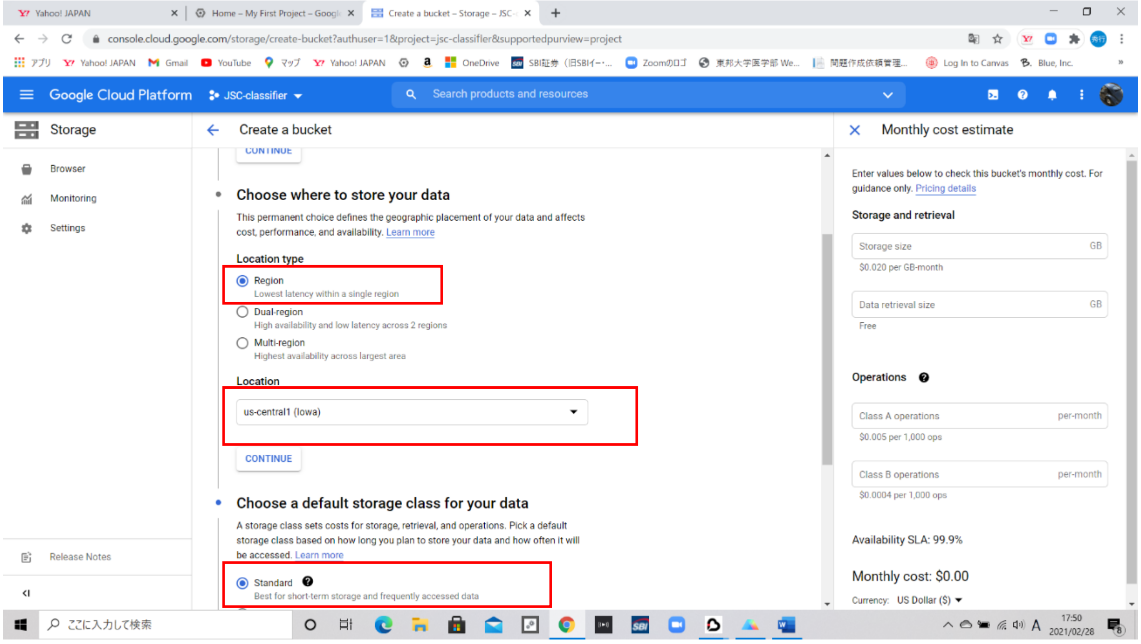

6. Create a dataset on Cloud AutoML Vision. Move to Cloud AutoML Vision from the GCP console. Create it at "Create new dataset". First, decide on the name of the dataset. For this type of research, check "Single-Label Classification" and click "CREATE DATASET". "Single-Label classification" is enclosed in a red frame.

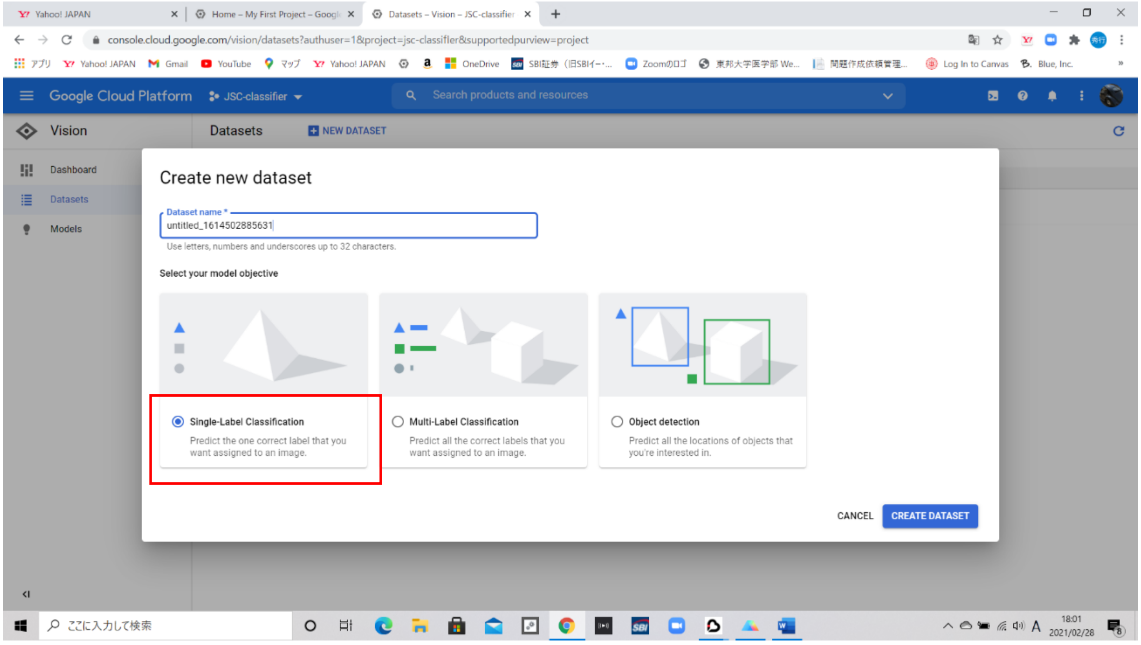

7. Make “Labels” on Cloud AutoML Vision. To make them, select the “IMAGES” tab and click “ADD NEW LABEL”. In this research, we made four labels, JSC_1_2_3, JSC_4_5, JSC_6_7, and JSC_8_9_10 as an initial dataset. “ADD NEW LABEL” is enclosed in a red frame.

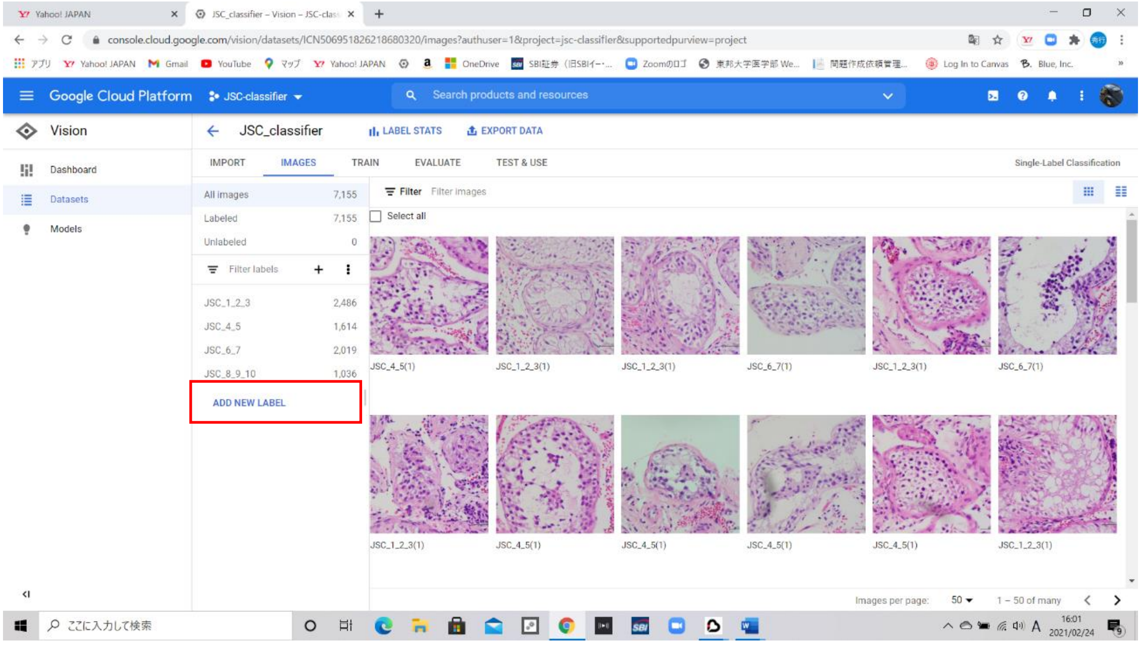

8. After finishing the above process, you can import images to Cloud AutoML Vision. In our research, we uploaded all data manually. We do not use csv files. To do this, check “Upload images from your computer”, click “SELECT FILES” under “Upload images from your computer” and then “BROWSE”. “SELECT FILES and “BROWSE” are enclosed in red frames.

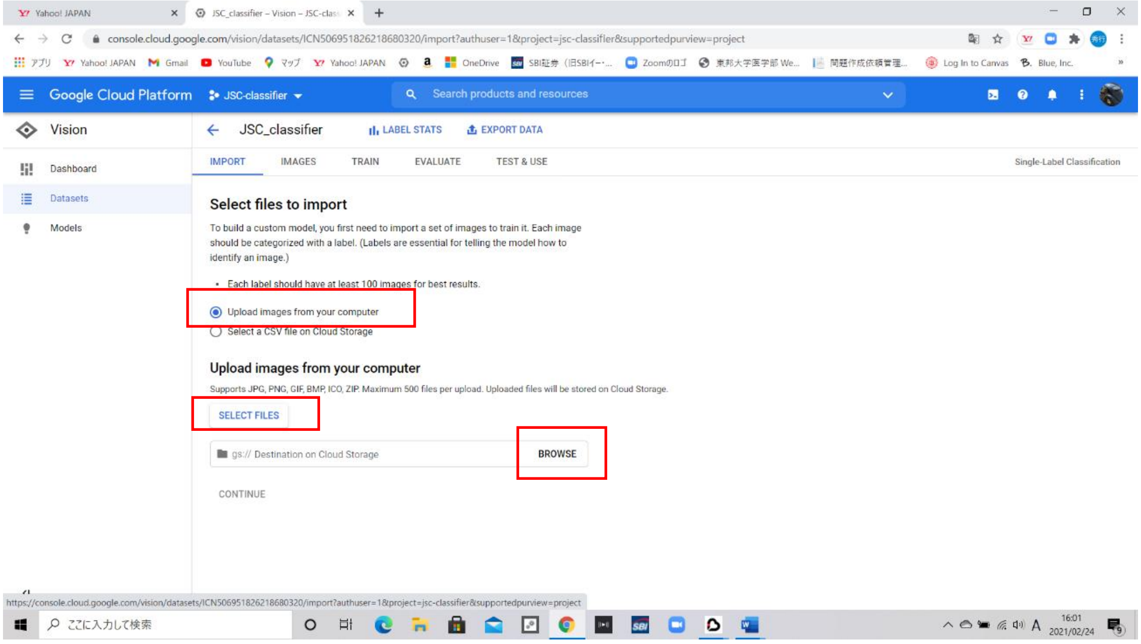

9. After clicking “BROWSE”, select the bucket you have already created. There is a point for attention. When you carry out the process for uploading images, you should upload more than ten images at one time. If you upload 9 images, all will become training data, not validation data and test data. In this research, the name of the bucket is “jsc_classifier”.
“jsc_classifier” is enclosed in a red frame.

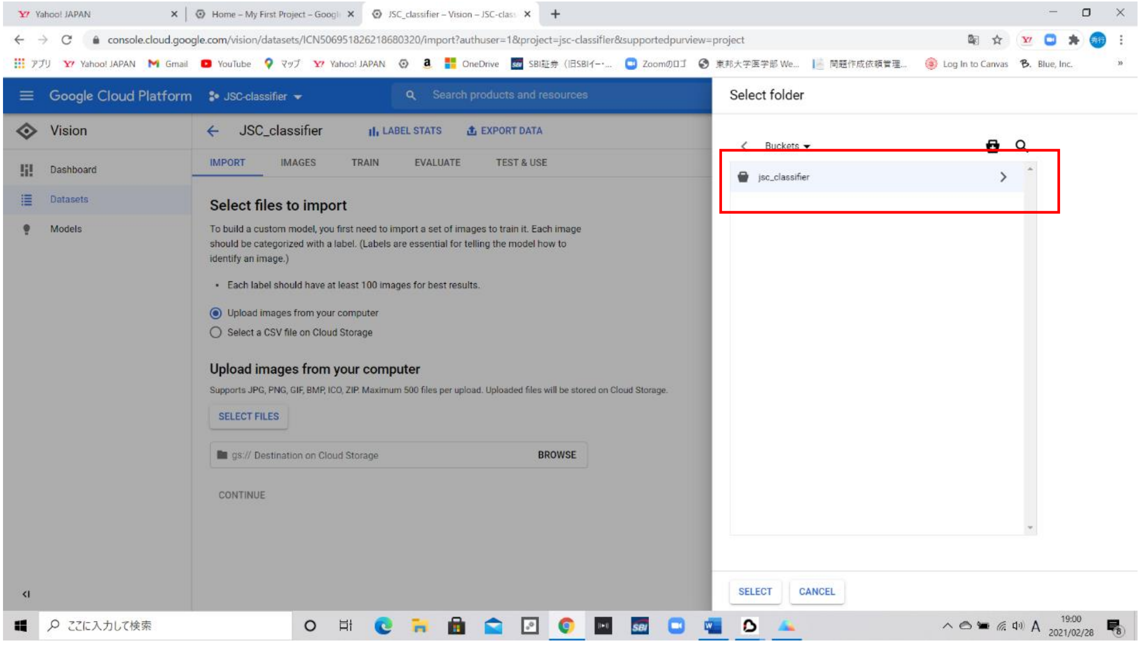

10. After finished the uploading process, click "Start training" under the "TRAIN" tab to start training automatically. In this research, we selected 8 nodes (1 hour) for the training time. In this screen, "Start training has changed to "TRAIN NEW MODEL" to perform retraining because we finished the training for our model in one go, " TRAIN NEW MODEL" is enclosed in a red frame.

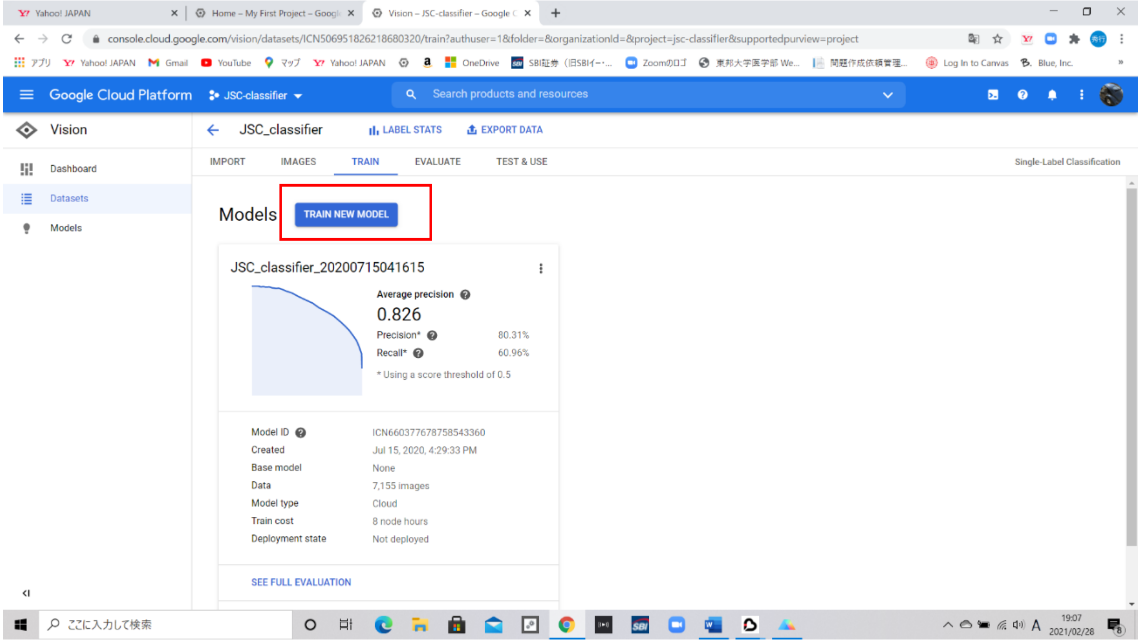

Supplement: Supplementary file 2 — Supplementary Information 2. [file 41598_2021_89369_MOESM2_ESM.pdf]
